# First Report on Cationic Triphenylphosphonium Compounds as Mitochondriotropic H_3_R Ligands with Antioxidant Properties

**DOI:** 10.3390/antiox13111345

**Published:** 2024-11-01

**Authors:** Tobias Werner, Tito Añazco, Paula Osses-Mendoza, Alejandro Castro-Álvarez, Cristian O. Salas, Raquel Bridi, Holger Stark, Christian Espinosa-Bustos

**Affiliations:** 1 Institute of Pharmaceutical and Medicinal Chemistry, Heinrich Heine University Düsseldorf, Universitaetsstr. 1, 40225 Duesseldorf, Germany; t.werner@hhu.de; 2 Departamento de Farmacia, Facultad de Química y de Farmacia, Pontificia Universidad Católica de Chile, Santiago 7820436, Chile; taanazco@uc.cl (T.A.); paula.osses@uc.cl (P.O.-M.); 3 Departamento de Ciencias Preclínicas, Facultad de Medicina, Universidad de La Frontera, Temuco 4780000, Chile; luis.castro@ufrontera.cl; 4 Departamento de Química Orgánica, Facultad de Química y de Farmacia, Pontificia Universidad Católica de Chile, Santiago 7820436, Chile; cosalas@uc.cl; 5 Departamento de Química Farmacológica y Toxicológica, Facultad de Ciencias Químicas y Farmacéuticas, Universidad de Chile, Santiago 8380000, Chile; raquelbridi@ciq.uchile.cl

**Keywords:** neurodegenerative diseases, antioxidants, histamine 3 receptor, triphenylphosphonium cation, molecular docking, molecular dynamics

## Abstract

Neurodegenerative diseases are a major public health problem due to the aging population and multifaceted pathology; therefore, the search for new therapeutic alternatives is of the utmost importance. In this sense, a series of six 1-(3-phenoxypropyl)piperidines alkyl-linked to a triphenylphosphonium cation derivative were synthesized as H_3_R ligands with antioxidant properties to regulate excessive mitochondrial oxidative stress and contribute to potential new therapeutic approaches for neurodegenerative diseases. Radioligand displacement studies revealed high affinity for H_3_R with K*i* values in the low to moderate two-digit nanomolar range for all compounds. Compound **6e** showed the highest affinity (K*i* H_3_R = 14.1 nM), comparable to that of pitolisant. Antioxidative effects were evaluated as radical-scavenging properties using the ORAC assay, in which all derivatives showed low to moderate activity. On the other hand, cytotoxic effects in SH-SY5Y neuroblastoma cells were investigated using the colorimetric alamar blue assay, which revealed significant effects on cell viability with an unequivocally structure–toxicity relationship. Finally, docking and molecular simulation studies were used to determine the H_3_R binding form, which will allow us to further modify the compounds to establish a robust structure-activity relationship and find a lead compound with therapeutic utility in neurodegenerative diseases.

## 1. Introduction

Neurodegenerative diseases like Alzheimer’s and Parkinson’s are characterized by progressive, irreversible neuronal loss, leading to a decrease in neurotransmitter levels and the formation of protein aggregates [1,2]. Although the causes and pathological characteristics of neurodegenerative diseases remain to be fully understood, iron imbalance, protein misfolding or aggregation, as well as mitochondrial dysfunction, have been identified as major mediators of oxidative stress and, as a consequence, neuronal cell death [3]. Normally, reactive oxygen species (ROS) play a key role in various physiological processes and are generally maintained in homeostasis by endogenous antioxidant systems [4]. However, when ROS production exceeds the endogenous antioxidant capacity, several detrimental mechanisms can be observed, which may lead to cell apoptosis [5]. Thus, mitochondria are key players in neurodegenerative disease pathogenesis, as they are one of the main sources as well as one of the critical ROS targets [6].

**Figure 1:**
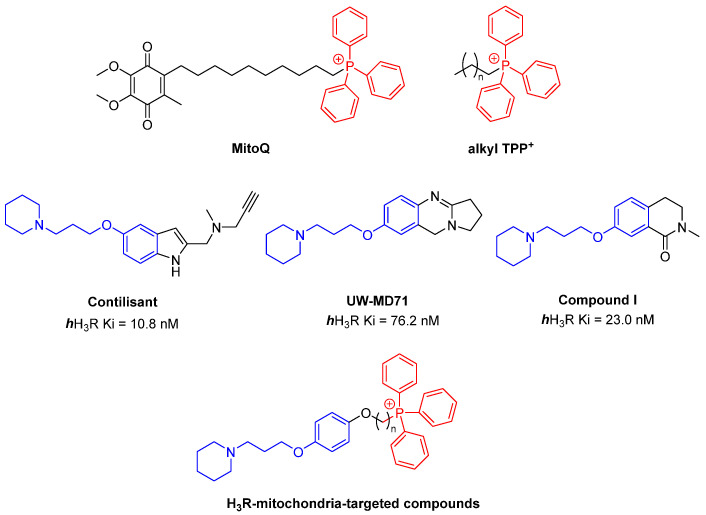
MitoQ, general alkyl TPP^+^, representative structures of H_3_R ligands, and target molecules for this study. Blue colour shows the H3 pharmacophore and red triphenylphosphonium scaffold.

Mitochondria are the central organelles of cells, with a unique structure consisting of two lipid-bilayer membranes that play essential roles in ATP synthesis, homeostasis of intracellular secondary messengers, and apoptosis [7,8]. It is well known that inadequate mitochondrial function can lead to increased susceptibility of neurons to oxidative stress and, consequently, neuronal death [6]. Given the important role of mitochondria in various cellular processes, compounds that target and accumulate within the mitochondria are of great therapeutic interest. In this field, the most common strategy for designing bioactive molecules to target mitochondria is to fuse a lipophilic cation via a linker to the pharmacophore, with the triphenylphosphonium (TPP^+^) scaffold being the most widely reported [9,10]. Many mitochondria-targeted drugs have been developed with TPP^+^ support, including Mito-Q (Figure 1), which has been widely used as an antioxidant to prevent cell death [11]. Benfeito et al. have developed multitarget directed mitochondriotropic antioxidants with promising neuroprotective properties that could be useful in the treatment of Alzheimer’s disease [12,13]. The accumulation of lipophilic cations in the mitochondrial matrix can modulate the mitochondrial membrane potential and affect mitochondrial function. Simple alkylated TPP^+^ cations have also been shown to inhibit mitochondrial respiration, and their potency is correlated with increasing hydrophobicity of the cation [14].

On the other hand, the histamine-3 receptor (H_3_R) is a member of the G protein-coupled receptor (GPCR) superfamily and has attracted considerable interest in the last few years due to its prevalence, primarily in the central nervous system, where it acts as an autoreceptor and heteroreceptor, regulating the release of histamine and other neurotransmitters such as dopamine and acetylcholine, among others [15,16]. It is known that there are several isoforms, most of which result from alternative splicing; however, functional and pharmacological implications of the complex isoform pattern remain underexplored [17]. Antagonists or inverse agonists of H_3_R prevent activation, thereby increasing histamine and other neurotransmitter levels through negative feedback. Therefore, they have been investigated as therapeutic alternatives for neurodegenerative diseases [18,19]. To date, there have been numerous reports of H_3_R ligands, all of which follow a well-described pharmacophore consisting of a basic unit separated from an aromatic region by a linker attached to an arbitrary scaffold that is typically hydrophobic [20]. Analyzing the structures of these ligands, there is a common fragment that is repeated in many of them, corresponding to 1-(3-phenoxypropyl)piperidine, which confers a great affinity for H_3_R. Contilisant (Figure 1) has been reported to exhibit H_3_R antagonism as well as inhibition of cholinesterase and monoamine oxidase B [21]. In 2014, Khan et al. presented new tetracyclic nitrogen-bridged compounds, where UW-MD71 showed affinity in the nanomolar range for H_3_R [22]. A series of lactams containing several basic scaffolds in their structure has been published by Dou et al., in which compound **I** showed high affinity for H_3_R and selectivity towards the H_1_R [23]. In 2021, Kuder et al. reported a set of H_3_R ligands that was designed to provide additional antioxidative effects [24].

Recognizing the urgent need for new neurodegenerative disease drugs and the multifactorial nature of these pathologies, in the present study, we designed, synthesized, and evaluated a new series of 1-(3-phenoxypropyl) piperidines linked to a triphenylphosphonium cation with several alkyl chains as multi-target compounds for potential use in the treatment of neurodegenerative diseases. Thus, our compounds could penetrate membranes and may move to/in mitochondria, which could potentially interfere with ROS production and simultaneously act as ligands at H_3_R, a well-established target for the treatment of neurodegenerative diseases.

## 2. Materials and Methods

### 2.1. Chemistry

Reagents and chemicals used in this work were purchased from Sigma Aldrich (St. Louis, MO, USA). Compounds that have been previously published were synthesized according to the respective procedures, as indicated with the relevant references. Purity of all synthesized compounds was determined by NMR, TLC, and HRMS. For TLC, silica gel 60 F254 25 aluminum foil 20 × 20 C (Merck, Burlington, CA, USA) was used as the stationary phase, and the mobile phase was specified in each reaction procedure. The chemical shifts in each signal in NMR spectra are reported in parts per million (ppm) and the coupling constants (*J*) in Hertz (Hz). NMR signal multiplicity was expressed as s (singlet), d (doublet), t (triplet), and dd (doublet doublet), as appropriate. The following instruments were used for the identification and characterization of each of the synthesized compounds: (1) Kofler Thermogerate apparatus (Reichert, Werke A.G., Wien, Vienna, Austria) for the determination of the melting points (mp), which are expressed in degrees Celsius (°C) without corrections; (2) BRUKER AVANCE III HD-400 spectrometers [400 MHz (^1^H) and 100 MHz (^13^C)] or 200 MHz [200 MHz (^1^H) and 50 MHz (^13^C)] to obtain the ^1^H and ^13^C NMR spectra (tetramethylsilane, TMS, was used as internal reference); and (3) BRUKER COMPACT MS + Elute SP LC with vacuum degasser, binary pump, autosampler, column oven at 40 °C, Intensity Solo 2 C18 column, and a constant nebulizer temperature of 220 °C, for the acquisition of HRMS-ESI data. This methodology was used for the final products.

#### 2.1.1. Synthesis of 1-(benzyloxy)-4-(3-bromopropoxy)benzene (**2**)

In a round-bottom flask with a magnetic stir bar, 4-(benzyloxy)phenol **1** (800 mg, 4.0 mmol) and potassium carbonate (1105 mg, 8.0 mmol) were added in 10 mL of acetonitrile. The mixture was stirred for 30 min, and then 1,3-dibromopropane was added and stirred at room temperature for 12 h. It was then filtered, and the solvent was removed under vacuum. The residue was purified by column chromatography on silica gel using hexane/ethyl acetate (4:1) as the eluent, resulting in a white solid, mp 53–55 °C, yield 75%. ^1^H NMR (400 MHz, acetone-*d*_6_) δ 7.32 (d, *J* = 7.4 Hz, 2H), 7.24 (t, *J* = 7.5 Hz, 2H), 7.17 (t, *J* = 7.2 Hz, 1H), 6.81 (dd, *J* = 9.4, 3.0 Hz, 2H), 6.75 (dd, *J* = 9.5, 3.5 Hz, 2H), 4.92 (s, 2H), 3.93 (t, *J* = 5.9 Hz, 2H), 3.52 (t, *J* = 6.6 Hz, 2H), 2.14 (p, *J* = 6.2 Hz, 2H). ^13^C NMR (101 MHz, acetone-*d*_6_) δ 153.21, 153.18, 137.81, 128.35 (2C), 127.63, 127.46 (2C), 115.75 (2C), 115.45 (2C), 70.09, 65.85, 32.47, 30.21.

#### 2.1.2. Synthesis of 1-(3-(4-(benzyloxy)phenoxy)propyl)piperidine (**3**)

Piperidine (0.28 mL, 2.81 mmol) was added to a magnetically stirred solution of 1-(benzyloxy)-4-(3-bromopropoxy)benzene **2** (600 mg, 1.87 mmol) and potassium carbonate (778 mg, 5.62 mmol) dissolved in acetonitrile (10 mL). The reaction mixture was then refluxed for 6 h, filtered, and the solvent evaporated under vacuum. The crude product was purified by column of silica gel using methylene chloride/methanol (20:1) as the eluent, resulting in a yellowish-white solid, mp 88–90 °C, yield 84%. ^1^H NMR (200 MHz, acetone-*d*_6_) δ 7.53–7.20 (m, 5H), 7.02–6.78 (m, 4H), 5.04 (s, 2H), 3.97 (t, *J* = 6.4 Hz, 2H), 2.54–2.33 (m, 6H), 1.98–1.81 (m, 2H), 1.63–1.42 (m, 6H). ^13^C NMR (50 MHz, acetone-*d*_6_) δ 154.47, 153.84, 138.74, 129.23 (2C), 128.50, 128.33 (2C), 116.60 (2C), 116.22 (2C), 71.00, 67.42, 56.33, 55.30 (2C), 27.68, 26.74 (2C), 25.23.

#### 2.1.3. Synthesis of 4-(3-(piperidin-1-yl)propoxy)phenol (**4**)

To a solution of **3** (450 mg, 1.38 mmol), Pd-C (35 mg) in ethanol (10 mL) with hydrogen atmosphere was added, and the mixture was stirred at room temperature for 24 h. After completion of the reaction, the mixture was filtered through celite and concentrated to give **4** as a white solid. 150–152 °C, yield 98%. ^1^H NMR (200 MHz, acetone-*d*_6_) δ 8.64 (s, 1H), 6.83–6.64 (m, 4H), 3.92 (t, *J* = 6.4 Hz, 2H), 2.56–2.26 (m, 6H), 1.98–1.78 (m, 2H), 1.65–1.35 (m, 6H). ^13^C NMR (50 MHz, CDCl_3_) δ 157.25, 156.93, 121.00 (2C), 120.66 (2C), 71.80, 60.65, 59.52 (2C), 31.90, 30.90 (2C), 29.43.

#### 2.1.4. General Procedure for the Preparation of Derivatives **5a**–**f**

Phenolic derivative **4** (1.0 equiv.) was dissolved in tetrahydrofuran (10 mL), and potassium carbonate (3.0 equiv.) was then added, and the mixture was stirred for 30 min. The corresponding dihaloalkane (1.5 equiv.) was added, and the reaction mixture was stirred at room temperature for 24 h. The solvent was then removed under reduced pressure, and the crude product was purified on a silica gel column using a 20:1 dichloromethane/methanol mixture as the mobile phase.

1-(3-(4-(4-Bromobutoxy)phenoxy)propyl)piperidine (**5a**). Yellowish-white oil, yield 67%. ^1^H NMR (400 MHz, CDCl_3_) δ 6.74 (s, 4H), 3.94 (t, *J* = 5.4 Hz, 2H), 3.88 (t, *J* = 5.6 Hz, 2H), 3.55 (t, *J* = 6.1 Hz, 2H), 2.96–2.90 (m, 6H), 2.28–2.21 (m, 2H), 2.01–1.81 (m, 8H), 1.55 (bs, 2H). ^13^C NMR (101 MHz, CDCl_3_) δ 153.34, 152.60, 115.45 (2C), 115.39 (2C), 67.59, 65.93, 55.51, 53.71 (2C), 44.78, 29.32, 26.73, 24.62, 23.34 (2C), 22.64.

1-(3-(4-((6-Bromopentyl)oxy)phenoxy)propyl)piperidine (**5b**). Yellow oil, yield 32%. ^1^H NMR (400 MHz, CDCl_3_) δ 6.81–6.65 (m, 4H), 3.93 (t, *J* = 5.6 Hz, 2H), 3.88–3.77 (m, 2H), 3.49 (t, *J* = 6.6 Hz, 2H), 3.06–2.87 (m, 5H), 2.27 (td, *J* = 11.3, 5.7 Hz, 2H), 2.01–1.81 (m, 5H), 1.83–1.66 (m, 4H), 1.63–1.50 (m, 4H). ^13^C NMR (101 MHz, CDCl_3_) δ 151.60, 150.59, 113.59 (2C), 113.49 (2C), 66.33, 63.92, 53.55, 51.70 (2C), 43.02, 30.42, 26.73, 22.49, 21.64, 21.18 (2C), 20.56.

1-(3-(4-((6-Bromohexyl)oxy)phenoxy)propyl)piperidine (**5c**). Yellow oil, yield 49%. ^1^H NMR (400 MHz, CDCl_3_) δ 6.79–6.68 (m, 4H), 3.95 (s, 2H), 3.89–3.78 (m, 2H), 3.48 (t, *J* = 6.7 Hz, 1H), 3.56–3.38 (m, 2H), 3.11 (bs, 2H), 2.63 (bs, 2H), 2.35 (bs, 2H), 2.29–2.17 (m, 2H), 1.91–1.71 (m, 8H), 1.53–1.28 (m, 6H). ^13^C NMR (101 MHz, CDCl_3_) δ 153.63, 152.29, 115.49 (2C), 115.37 (2C), 68.35, 65.61, 55.43, 53.51 (2C), 45.01, 32.49, 29.16, 26.62, 25.38, 23.98, 22.58 (2C), 22.12.

1-(3-(4-((8-Bromooctyl)oxy)phenoxy)propyl)piperidine (**5d**). Yellow oil, yield 90%. ^1^H NMR (400 MHz, acetone-*d*_6_) δ 6.93–6.80 (m, 4H), 4.05 (t, *J* = 6.2 Hz, 2H), 3.93 (t, *J* = 6.5 Hz, 2H), 3.12–3.05 (m, 6H), 2.35–2.21 (m, 2H), 2.09–2.04 (m, 2H), 1.96–1.87 (m, 2H), 1.88–1.78 (m, 10H), 1.74 (dd, *J* = 14.3, 6.8 Hz, 2H), 1.69–1.57 (m, 2H), 1.39 (dd, *J* = 7.2, 3.7 Hz, 2H). ^13^C NMR (101 MHz, acetone-*d*_6_) δ 153.52, 152.91, 115.48 (2C), 115.26 (2C), 68.09, 66.12, 54.56, 52.91 (2C), 48.73, 33.98, 32.70, 27.84, 25.79, 24.41, 23.69 (2C), 23.24, 22.34, 19.50.

1-(3-(4-((9-Bromononyl)oxy)phenoxy)propyl)piperidine (**5e**). Yellow oil, yield 82%. ^1^H NMR (400 MHz, acetone-*d*_6_) δ 6.76–6.68 (m, 4H), 3.90 (t, *J* = 6.1 Hz, 2H), 3.79 (t, *J* = 6.5 Hz, 2H), 3.36 (t, *J* = 6.8 Hz, 2H), 2.89–2.79 (m, 6H), 2.15–2.05 (m, 2H), 1.78–1.70 (m, 6H), 1.65–1.56 (m, 2H), 1.50–1.44 (m, 2H), 1.37–1.30 (m, 4H), 1.27–1.20 (m, 6H). ^13^C NMR (101 MHz, acetone-*d*_6_) δ 153.53, 152.93, 115.43, 115.26, 68.10, 66.13, 54.70, 53.12 (2C), 33.94, 32.73, 29.22, 27.88, 25.85, 23.49 (2C), 22.61.

1-(3-(4-((10-Bromodecyl)oxy)phenoxy)propyl)piperidine (**5f**). Yellow oil, yield 87%. ^1^H NMR (200 MHz, CDCl_3_) δ 6.74 (s, 4H), 3.85 (dt, *J* = 13.2, 6.4 Hz, 4H), 3.33 (t, *J* = 6.8 Hz, 2H), 2.60–2.38 (m, 4H), 1.97 (dd, *J* = 14.9, 6.5 Hz, 2H), 1.85–1.50 (m, 8H), 1.46–1.10 (m, 16H). ^13^C NMR (50 MHz, CDCl_3_) δ 153.32, 152.95, 115.40 (4C), 68.61, 66.92, 55.94, 54.42 (2C), 34.00, 32.81, 29.41, 29.35, 29.33 (2C), 28.72, 28.14, 26.43, 26.01, 25.39 (2C), 24.05.

#### 2.1.5. General Procedure for the Preparation of Derivatives **6a**–**f**

A solution of the respective **5a**–**f** derivative (1.0 eq.) and triphenylphosphine (2.0 eq.) in acetonitrile (5 mL) was refluxed for 48–72 h. After completion of the reaction, the solvent was removed, and the crude product was purified by silica gel chromatography using methylene chloride/methanol (10:1) as the eluent to afford intermediate **6a**–**f**.

Triphenyl-(5-(4-(3-(piperidin-1-yl)propoxy)phenoxy)butyl)phosphonium bromide (**6a**). Yellow oil, yield 44%. ^1^H NMR (400 MHz, CDCl_3_) δ 7.75–7.70 (m, 9H), 7.66–7.58 (m, 6H), 6.70 (d, *J* = 10.4 Hz, 2H), 6.63 (d, *J* = 8.6 Hz, 2H), 3.91 (q, *J* = 5.8 Hz, 4H), 3.70 (dd, *J* = 15.7, 12.9 Hz, 2H), 3.03–2.90 (m, 6H), 2.20 (dt, *J* = 11.3, 5.6 Hz, 2H), 2.12–2.02 (m, 2H), 1.90–1.72 (m, 6H), 1.56 (b.s., 2H). ^13^C NMR (101 MHz, CDCl_3_) δ 152.84, 152.78, 135.16 (d, *^4^J_C-P_* = 2.9 Hz, 3C), 133.60 (d, *^3^J_C-P_* = 10.0 Hz, 6C), 130.54 (d, *^2^J_C-P_* = 12.5 Hz, 6C), 118.11 (d, *^1^J_C-P_* = 86.0 Hz, 3C), 115.56, 115.33, 66.80, 66.10, 55.30, 53.58 (2C), 29.28 (d, *^3^J_C-P_* = 16.7 Hz), 25.94, 24.81, 23.65 (2C), 22.52, 22.33 (d, *^1^J_C-P_* = 50.9 Hz), 21.83, 19.36 (d, *^2^J_C-P_* = 4.0 Hz). ^31^P NMR (162 MHz, CDCl_3_) δ 24.27. LC/HR-MS for (C_35_H_41_NO_2_P^+^ [M^+^]), Calcd: 552.3026, Found: 552.3037.

Triphenyl-(5-(4-(3-(piperidin-1-yl)propoxy)phenoxy)pentyl)phosphonium bromide (**6b**). Yellow oil, yield 73%. ^1^H NMR (400 MHz, acetone-*d*_6_) δ 8.03–7.89 (m, 9H), 7.83–7.76 (m, 6H), 6.89 (d, *J* = 9.0 Hz, 2H), 6.78 (d, *J* = 9.0 Hz, 2H), 4.08 (t, *J* = 6.1 Hz, 2H), 3.91 (b.s., 2H), 3.84–3.73 (m, 2H), 3.56 (d, J = 10.1 Hz, 2H), 3.34–3.24 (m, 2H), 2.42 (dt, *J* = 13.4, 6.2 Hz, 2H), 2.31–2.19 (m, 2H), 1.96–1.74 (m, 10H). ^13^C NMR (101 MHz, acetone-*d*_6_) δ 153.35, 152.86, 135.05 (d, *^4^J_C-P_* = 2.9 Hz, 3C), 133.87 (d, *^3^J_C-P_* = 10.0 Hz, 6C), 130.36 (d, *^2^J_C-P_* = 12.5 Hz, 6C), 118.83 (d, *^1^J_C-P_* = 86.1 Hz, 3C), 115.59, 115.34, 78.43, 67.81, 66.02, 54.24, 52.45, 29.72, 28.22, 27.01 (d, *^3^J_C-P_* = 16.8 Hz), 23.62, 22.37, 22.04 (d, *^2^J_C-P_* = 4.3 Hz), 21.80, 21.50 (d, *^1^J_C-P_* = 41.5 Hz). ^31^P NMR (162 MHz, acetone-*d*_6_) δ = 24.11. LC/HR-MS for (C_37_H_45_NO_2_P^+^ [M^+^]), Calcd: 566.3182, Found: 566.3185.

Triphenyltriphenyl-(6-(4-(3-(piperidin-1-yl)propoxy)phenoxy)hexyl)phosphonium bromide (**6c**). Yellow oil, yield 94%. ^1^H NMR (400 MHz, CDCl_3_) δ 7.82–7.46 (m, 15H), 6.71–6.49 (m, 4H), 3.85 (t, *J* = 5.8 Hz, 2H), 3.71 (t, *J* = 6.2 Hz, 2H), 3.52–3.39 (m, 2H), 3.20–3.12 (m, 4H), 2.26 (td, *J* = 11.4, 5.8 Hz, 2H), 1.91 (bs, 2H), 1.63–1.44 (m, 8H), 1.38–1.28 (m, 2H), 1.22–1.02 (m, 4H). ^13^C NMR (101 MHz, CDCl_3_) δ 153.28, 152.40, 135.22 (d, *^4^J_C-P_* = 2.9 Hz, 3C), 133.48 (d, *^3^J_C-P_* = 9.9 Hz, 6C), 130.58 (d, *^2^J_C-P_* = 12.5 Hz, 6C), 118.01 (d, *^1^J_C-P_* = 86.0 Hz, 3C), 115.46, 115.38, 68.11, 65.65, 54.98, 53.11, 30.00 (d, *^3^J_C-P_* = 15.9 Hz), 28.76, 25.46, 24.00, 22.71 (d, *^1^J_C-P_* = 43.4 Hz), 22.46 (d, *^2^J_C-P_* = 4.4 Hz), 22.41, 21.73. ^31^P NMR (162 MHz, CDCl3) δ 23.91. LC/HR-MS for (C_38_H_47_NO_2_P^+^ [M^+^]), Calcd: 580.3339, Found: 580.3332.

Triphenyltriphenyl-(8-(4-(3-(piperidin-1-yl)propoxy)phenoxy)octyl)phosphonium bromide (**6d**). Yellow oil, yield 19%. ^1^H NMR (200 MHz, CDCl_3_) δ 7.92–7.63 (m, 15H), 6.79 (s, 4H), 3.97 (t, *J* = 6.0 Hz, 2H), 3.86 (t, *J* = 6.4 Hz, 2H), 2.83 (bs, 14H), 2.26–2.07 (m, 2H), 1.89–1.76 (m, 4H), 1.74–1.44 (m, 8H). ^13^C NMR (50 MHz, CDCl_3_) δ 153.30, 152.67, 135.18 (d, *^4^J_C-P_* = 3.0 Hz, 3C), 133.47 (d, *^3^J_C-P_* = 9.9 Hz, 6C), 130.55 (d, *^2^J_C-P_* = 12.5 Hz, 6C), 118.10 (d, *^1^J_C-P_* = 86.0 Hz, 3C), 115.52 (2C), 115.40 (2C), 68.45, 66.36, 55.56, 53.85, 29.01 (d, *^3^J_C-P_* = 11.1 Hz), 28.82, 25.78, 25.28, 24.15, 23.12–22.12 (d, *^1^J_C-P_* = 45.0 Hz), 23.02, 22.50 (d, *^2^J_C-P_* = 4.4 Hz). ^31^P NMR (162 MHz, CDCl_3_) δ 23.96. LC/HR-MS for (C_40_H_51_NO_2_P^+^ [M^+^]), Calcd: 608.3652, Found: 608.3663.

Triphenyltriphenyl-(9-(4-(3-(piperidin-1-yl)propoxy)phenoxy)nonyl)phosphonium bromide (**6e**). Yellow oil, yield 24%. ^1^H NMR (400 MHz, acetone-*d*_6_) δ 8.04 (dd, *J* = 12.6, 7.7 Hz, 6H), 7.93 (t, *J* = 7.4 Hz, 3H), 7.80 (td, *J* = 7.7, 3.2 Hz, 6H), 6.93–6.83 (m, 4H), 4.02 (t, *J* = 6.2 Hz, 2H), 3.98–3.85 (m, 4H), 3.01 (b.s., 6H), 2.27–2.16 (m, 2H), 1.84 (d, *J* = 5.0 Hz, 4H), 1.78–1.53 (m, 8H), 1.46–1.28 (m, 8H). ^13^C NMR (101 MHz, acetone-*d*_6_) δ 153.44, 152.95, 134.92 (d, *^4^J_C-P_* = 3.0 Hz, 3C), 133.99 (d, *^3^J_C-P_* = 10.1 Hz, 6C), 130.30 (d, *^2^J_C-P_* = 12.5 Hz, 6C), 119.03 (d, *^1^J_C-P_* = 85.9 Hz, 3C), 115.54 (2C), 115.30 (2C), 68.10, 66.24, 54.58, 53.01, 30.04 (d, *^3^J_C-P_* = 16.3 Hz), 29.15, 28.97 (2C), 28.57, 25.78 (2C), 24.77, 23.64 (2C), 22.58, 22.27 (d, *^2^J_C-P_* = 4.3 Hz), 21.57 (d, *^1^J_C-P_* = 49.9 Hz). ^31^P NMR (162 MHz, acetone-*d*_6_) δ = 24.31. LC/HR-MS for (C_41_H_53_NO_2_P^+^ [M^+^]), Calcd: 622.3808, Found: 622.3813.

Triphenyltriphenyl-(10-(4-(3-(piperidin-1-yl)propoxy)phenoxy)decyl)phosphonium bromide (**6f**). Yellow oil, yield 93%. ^1^H NMR (400 MHz, CDCl_3_) δ 7.84–7.56 (m, 15H), 6.72 (s, 4H), 3.92 (t, *J* = 5.5 Hz, 2H), 3.80 (t, *J* = 6.3 Hz, 2H), 3.46 (b.s., 2H), 3.09 (b.s., 4H), 2.38–2.19 (m, 2H), 1.92 (b.s., 4H), 1.64–1.54 (m, 8H), 1.38–1.26 (m, 2H), 1.18 (b.s., 10H). ^13^C NMR (101 MHz, CDCl_3_) δ 153.48, 152.50, 135.27 (d, *^4^J_C-P_* = 3.0 Hz, 3C), 133.61 (d, *^3^J_C-P_* = 10.1 Hz, 6C), 130.64 (d, *^2^J_C-P_* = 12.5 Hz, 6C), 118.02 (d, *^1^J_C-P_* = 85.9 Hz, 3C), 115.53 (2C), 115.47 (2C), 68.55, 65.98, 55.24, 53.42 (2C), 30.43 (d, *^3^J_C-P_* = 15.5 Hz), 29.67, 29.28, 29.22, 29.14, 29.01 (2C), 25.89, 24.61, 23.15 (d, *^1^J_C-P_* = 50.5 Hz), 23.30, 22.55 (d, *^2^J_C-P_* = 4.5 Hz), 22.20. ^31^P NMR (162 MHz, CDCl_3_) δ 24.05. LC/HR-MS for (C_42_H_55_NO_2_P^+^ [M^+^]), Calcd: 636.3965, Found: 636.3964.

### 2.2. hH_3_R Radioligand Displacement Assay

Radioligand displacement assays were performed on the membrane fractions of HEK-293 cells stably expressing hH_3_R_455_, which occurs mainly in the brain, as described previously with slight modifications [25]. HEK-293-hH_3_R cells were grown in Dulbecco’s Modified Eagle Medium (DMEM) supplemented with 10% FBS, 100 µg/mL penicillin, 100 units/mL streptomycin, and 10 mM HEPES solution at 37 °C in a humidified atmosphere of 5% CO_2_. To prepare membrane fractions, cells were washed in a phosphate-buffered saline (PBS) solution and harvested. The cells were centrifuged at 3000× *g* for 10 min at 4 °C and homogenized with Ultraturrax^®^ in ice-cold binding buffer (75 mM Tris-HCl, 10 mM MgCl_2_, 100 mM NaCl, pH 7.4). The homogenate was centrifuged twice (20,000× *g*, 20 min, 4 °C) and reconstituted in an ice-cold binding buffer. Test ligands, radiolabeled ligands, and membrane fractions (20 µg/well) were added to 96-well plates to a final assay volume of 200 µL. After 90 min of incubation with shaking at 25 °C, a cell harvester was used to separate bound from free ligands by filtration through GF/B filters pretreated with 0.3% (*m*/*v*) polyethylenimine. Radioactivity was determined by liquid scintillation counting. The radiolabeled probe [^3^H]*N*^α^-methylhistamine was used at a final concentration of 2 nM (K*d* = 3.08 nM). The test ligands were evaluated at concentrations between 100 µM and 0.03 nM. To determine non-specific binding, 10 µM pitolisant was used. Specific binding was calculated by subtracting non-specific binding from the raw data. Data were obtained in duplicate from at least three independent experiments and analyzed using GraphPad Prism 7.02. using a nonlinear regression. The Cheng–Prusoff equation was used to calculate K*i* values from the IC_50_ values. Mean values and 95% confidence intervals were calculated using −log(K*_i_*) and converted to nanomolar concentrations.

### 2.3. Cytotoxic Effects on SH-SY5Y Cells After 24 h

The cytotoxic effects of the tested ligands on neuroblastoma SH-SY5Y cells were determined using the alamar blue viability assay [26]. SH-SY5Y cells were grown in Minimum Essential Medium Eagle (MEME) supplemented with non-essential amino acids, 10% FBS, 100 µg/mL penicillin, 100 units/mL streptomycin, and 2 mM L-glutamine at 37 °C in a humidified atmosphere of 5% CO_2_. SH-SY5Y cells were seeded in 96-well plates at a density of 8.0 × 10^3^ cells per well and grown at 37 °C in a humidified atmosphere of 5% CO_2_. 24 h after seeding, the test ligands were added at concentrations between 30 µM and 1 nM in a final assay volume of 100 µL per well and incubated for 24 h. Cells treated with 1-methyl-4-phenylpyridinium iodide (MPP+) served as a positive control for cytotoxic effects. The concentration of DMSO was limited to 1% for all the assay points. The medium supplemented with the test compounds was discarded, and 100 µL of resazurin solution in RPMI-1640 medium (44 µM) was added. After two hours of incubation at 37 °C in a humidified atmosphere of 5% CO_2_, fluorescence intensity (λ_Em_ = 535 nm, λ_Ex_ = 590 nm) was determined. Data were obtained in duplicate from at least three independent experiments. LC_50_ values were evaluated using GraphPad Prism 7.02. using a nonlinear regression. Mean values and 95% confidence intervals were calculated using −log(K*i*) and converted to nanomolar concentrations.

### 2.4. Antioxidant Properties

Antioxidant properties were analyzed using an oxygen radical absorbance capacity (ORAC) assay at 37 °C [27]. Solutions of the fluorescent probe fluorescein at 100 nM and test ligands at concentrations between 20 µM and 6.25 µM were incubated for 15 min in potassium phosphate buffer (75 mM, pH 7.4) protected from light in black 96-well plates at 37 °C. Then, 2,2′-azobis (2-amidinopropane) dihydrochloride (AAPH) was added as a peroxyl radical generator. The fluorescence intensity (λ_Em_ = 492 nm, λ_Ex_ = 516 nm) was measured every minute for 90 min. 6-Hydroxy-2,5,7,8-tetramethylchroman-2-carboxylic acid (Trolox) was used as a standard at concentrations between 20 µM and 6.25 µM. Data were obtained in duplicate from at least three independent experiments and evaluated using GraphPad Prism 7.02. Fluorescence intensity was normalized to that of the blank (without AAPH). The area under the fluorescence decay curve for each antioxidant (AUC_antioxidant_) and for the negative control without antioxidants (AUC_negative control_) were calculated for each concentration by summing the fluorescence intensities at each time point. AUC_negative control_ was subtracted from the AUC_antioxidant_ to calculate the net AUC. Linear regression of the net AUC against the corresponding antioxidant concentration was performed. The ratios of the antioxidant and Trolox slopes from the same experiment were calculated. Thus, ORAC values were expressed as Trolox equivalents [TE], and caffeic acid was used as a reference [28].

### 2.5. Simulation Ligand–Protein System

#### 2.5.1. Ligand and Protein Preparation

The ligands were created using Avogadro software v. 1.2 to obtain the three-dimensional chordates of each of the ligands (**6a**–**6f**), assigning the corresponding aromaticity and hydrogens. Subsequently, partial charges were assigned using the QUACPAC program v. 2.1.3, and protonations were generated using FixPka v. 2.1.3. The conformations of each ligand were obtained using Omega v. 4.1.2.0 software [29].

For H_3_R, three-dimensional coordinates available in the Protein Data Bank (PDB ID: 7F61) were used [30]. Missing amino acid residues were added to the Prime program v. 7.5, water molecules were removed, and hydrogen atoms were incorporated using the Protein Preparation module of the Schrödinger Suite v. 2024-1. In addition, the respective protonations for basic and acidic amino acid residues were assigned at pH 7.32 using PropKa v. 3.0, and the orientation of the side chains of the residues was adjusted using the OPLS4 force field [31].

#### 2.5.2. Induced-Fit Docking

Because of the size of the synthesized ligands, induced-fit docking (IFD) was performed considering the center of mass of the co-crystallized ligand of the 7F61 crystal (ligand PF03654746) as the center of the grid for molecular docking at 20 Å. A constraint was maintained for the electrostatic interaction with the Asp114 binding site, and an energy window of 2.5 kcal/mol was maintained. The residues and backbone of any amino acid located 5.0 Å from the ligand positioned at the binding site were relaxed. The SP scoring protocol was used to estimate the affinity energy for each docked ligand.

#### 2.5.3. Molecular Dynamics

Ligand–protein complexes with higher energies were subjected to molecular dynamics to evaluate the predominant interactions. The system preparation included OPLS4 force-field assignment and explicit solvent model TIP3P, considering a 0.15 M NaCl concentration. In addition, Charmm-Gui was used to add a lipid bilayer using POPC to both sides of the layer. Once the complex with explicit solvation was obtained (approximately 41,000 total atoms), molecular dynamics (MD) simulations were performed under NPT conditions (constant number of particles, pressure, and temperature), maintaining a constant temperature of 300 K and a pressure of 1.013 bar. The equations of motion were calculated using the RESPA integrator with an internal time step of 2.0 fs for bound and unbound interactions within the short-range cutoff. Nose-Hoover thermostats were applied to keep the temperature constant and the Martyna-Tobias-Klein method to control the pressure. The limit for van der Waals and short-range electrostatic interactions was set at 9.0 Å, and long-range electrostatic interactions were calculated using the particle-mesh Ewald (PME) method. To ensure system equilibrium, a predetermined protocol was used to ensure system equilibrium, which included a series of constrained minimizations and MD simulations to relax the system gradually. The trajectories were extended to 100 ns, and the analysis was performed using Python scripts v. 3.9.

## 3. Results and Discussion

### 3.1. Chemistry

**Scheme 1:**
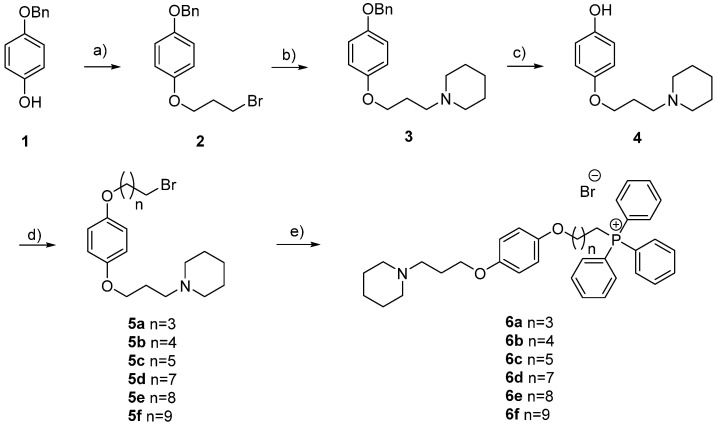
Reagents and conditions. (**a**) 1,3-Dibromopropane, K_2_CO_3_, CH_3_CN, r.t., 12 h, 75%. (**b**) Piperidine, K_2_CO_3_, CH_3_CN, reflux, 6 h, 84%. (**c**) H_2_, Pd/C 10%, ethanol, r.t. 24 h, 98%. (**d**) α,ω-Dibromoalkanes, K_2_CO_3_, THF, r.t., 24 h, 32–90%. (**e**) Triphenylphosphine, CH_3_CN, reflux, 48–72 h, 19–94%.

The synthesis of triphenyl(2-(4-(3-(piperidin-1-yl)propoxy)phenoxy)alkyl)phosphonium derivatives **6a**–**f** was carried out according to the synthetic route shown in Scheme 1. From 4-(benzyloxy)phenol **1** and through an *O*-alkylation reaction with 1,3-dibromopropane and K_2_CO_3_ in acetonitrile, 1-(benzyloxy)-4-(3-bromopropoxy)benzene **2** was obtained in 75% yield [32]. Subsequently, via the S_N_2 reaction, the piperidine moiety was added to the basic medium, resulting in derivative **3** in good yield (84%) [32]. To remove the benzyl group, catalytic hydrogenation was performed using Pd/C 10% in ethanol as the solvent, yielding 4-(3-(piperidin-1-yl)propoxy)phenol **4** in a quantitative yield. In the next step of the synthetic route, derivative **4** was alkylated with different α,ω-dibromoalkanes by removing the acidic proton from phenol using potassium carbonate in tetrahydrofuran, resulting in derivatives **5a**–**f** with yields of 32–90%. Finally, by addition of an excess of triphenylphosphine in acetonitrile under reflux for two days, the final derivatives **6a**–**f** were obtained [33]. The characterization and assignment of the signals of our compounds were determined by ^1^H and ^13^C NMR and for the final derivatives **6a**–**f**, ^1^H, ^13^C, ^31^P, COSY, HSQC, HMBC, and mass spectrometry. The purity of the final compounds was determined by HPLC (see Supplementary Materials).

### 3.2. Biological Evaluation

H_3_R affinities of target compounds **6a**–**f** were determined in [^3^H]Nα-methylhistamine radioligand displacement studies (Table 1) using crude membrane extracts from hH_3_R-HEK293 cells [34]. In general, all the compounds in this work presented affinity values (K*i*) in the low nanomolar range; moreover, compound **6e** presented a value of K*i* = 14.1 nM, which is comparable to the affinity of pitolisant (K*i* = 13.1 nM), a reference drug approved by the FDA and EMA for the treatment of narcolepsy [35]. Length variation in the alkyl chain did not lead to significantly different affinities towards H_3_R. However, derivative **6f** had the lowest affinity (K*i* = 67.4 nM) and the longest chain with ten carbon atoms, which could be due to increased non-specific binding but remains the subject of future investigations.

As these compounds were developed to also accumulate in the mitochondria to prevent ROS damage, their radical-scavenging properties were investigated. Compounds **6a**–**f** were evaluated by determining the oxygen radical absorbance capacity (ORAC) using the ORAC-Fluorescein (ORAC-FL) method. [27]. Radical-scavenging properties of the compounds were expressed as Trolox equivalents (TE) using caffeic acid as a reference (Table 1). From the point of view of the structure-activity relationship, no structural features were incorporated that could be expected to have major antioxidant effects. However, all compounds showed low oxygen radical absorbance capacity ranging from 0.54 TE for **6c** to 1.19 TE for **6d**, while the compounds had no clear lag time before the decay of fluorescein. This observation could be attributed to lower reactivity compared to the fluorescein probe, in which case the ORAC-FL method tends to overestimate the effect of weak antioxidants [36]. On the other hand, we have to state that we could not follow the fate of the compound if it is trapped in a specific membrane region or taken for catabolic reactions and therefore, avoid raising hypotheses, which are not data driven.

**Figure 2:**
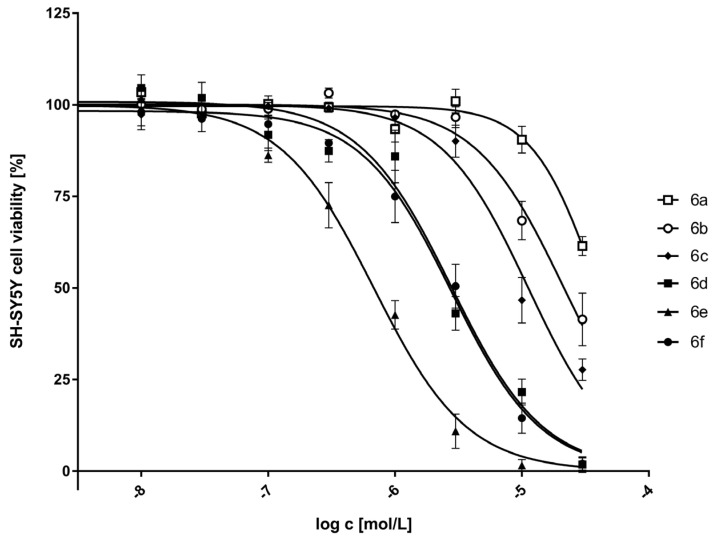
Viability of SH-SY5Y cells upon treatment with derivatives **6a**–**f**.

The in vitro cytotoxicity of derivatives **6a**–**f** was evaluated in the human neuroblastoma cell line SH-SY5Y (Table 1). This cell line is extensively used in neurotoxicity studies and represents a model for neurodegenerative diseases [32]. Compound **6e** exhibited an LC_50_ value below 1 µM; all other compounds were in the micromolar range. In contrast to previous assays, significant effects on the viability of SH-SY5Y cells for the different alkyl chain lengths were recognized. As the alkyl chain length increased, the LC_50_ decreased, establishing a clear structure–toxicity relationship that can be used for future optimization approaches (Figure 2). Increased alkyl chain length comes with increased hydrophobicity, leading to non-specific binding to the inner mitochondrial membrane and thus to disruption of membrane integrity [12]. Therefore, limiting the alkyl chain length seems to be of crucial importance to achieve a favorable balance between activity and safety for the ligand design. However, it is important to note that the affinities for H_3_R were in the low two-digit nanomolar range, and compounds **6a** and **6b** showed no significant effects on cell viability at concentrations below 1 µM.

### 3.3. Molecular Docking

The synthesized ligands were positioned in the binding pocket of H_3_R protein (PDB ID: 7F61). Since this protein was co-crystallized with compound PF03654746, docking with induced fit was performed, considering a radius of 5.0 Å around the ligand to correct the side chains of the amino acid residues composing the binding site to properly position each of the synthesized ligands.

**Figure 3:**
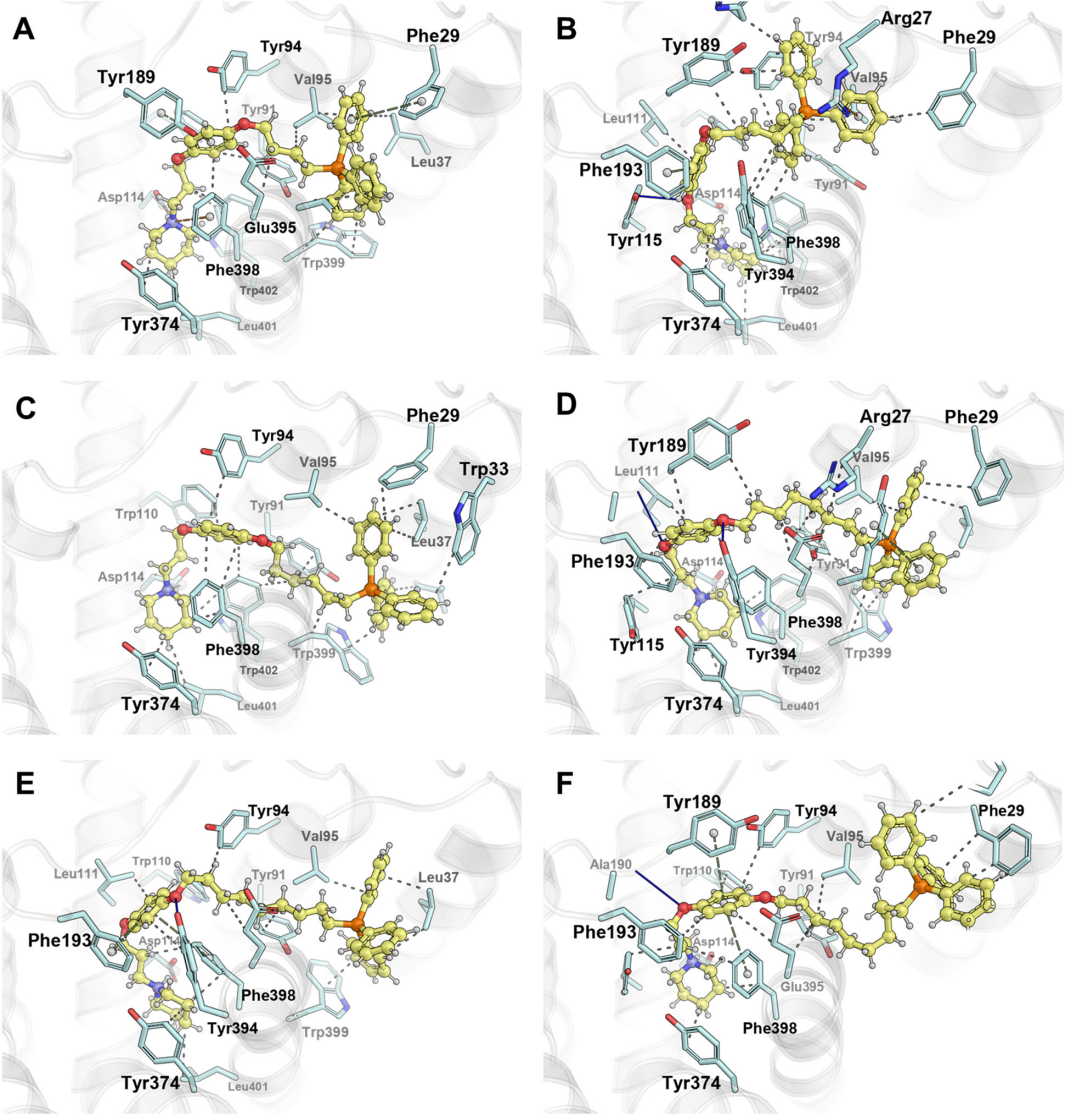
Molecular docking of synthesized ligands in the binding pocket of H_3_R protein. (**A**) **6a**, (**B**) **6b**, (**C**) **6c**, (**D**) **6d**, (**E**) **6e**, and (**F**) **6f**.

These results are presented in Figure 3 and show a correlation between the affinity energy and biological activity (Table 2). All ligands presented electrostatic interaction between the Asp114 residue and the protonated piperidine, which was facilitated by the restriction imposed by the IFD method, which oriented the central benzyl ring towards the amino acid residues of Tyr39, Tyr94, Tyr189, and Phe193. The extension of the alkyl chain provided various orientations of phosphine between the amino acid residues of Phe29, Arg27, Trp33, Leu37, Val95, and Trp399, generating hydrophobic and π-stacking interactions.

**Figure 4:**
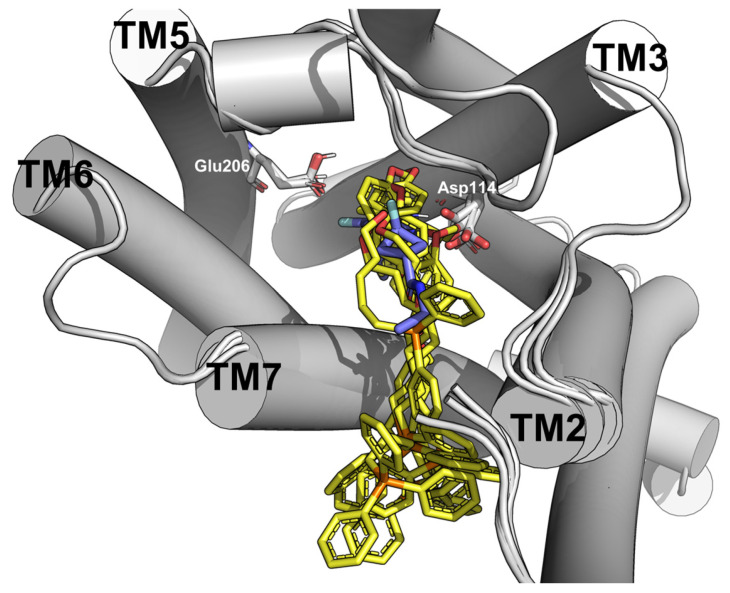
IFD orientation of the synthesized ligands (yellow) compared to the co-crystallized ligand (purple).

One reason for the biological activity of alkyl chain extension is that phosphine acts as a blocking fragment at the binding site. The presence of a ligand with a four-carbon chain extension (4C) hinders the interaction of the tertiary amine with Asp114 residue. In contrast, compound **6b** showed a better reach, allowing phosphine to avoid interference with electrostatic interactions. As the extent of the linker increased, phosphine was accommodated between the transmembrane (TM) TM2 and TM7 domains (Figure 4), which contain aromatic residues (Phe29, Trp33, and Trp399). However, chain length complicates the correct π–π interactions. The linker extension of compound **6e** was ideal for the interaction with the Asp114 residue (internal pocket) and the aromatic residues of TM2 and TM7. However, compound **6f** exceeded the tolerated distance, and the connector had too many degrees of freedom, which impaired the hydrophobic interactions.

### 3.4. Molecular Dynamics

Molecular dynamics were performed with compounds **6b** and **6e**, which were considered the most active, and compounds **6c** and **6f**, which were considered the least active, with the aim of identifying the predominant elements in a system with explicit solvation. This system includes a lipid bilayer to establish the necessary rigidity of the transmembrane domains of the H_3_R protein. Molecular dynamics simulations were performed for 100 ns, and analyses of protein–ligand contacts were performed with the Simulation Interactions Diagram module of Desmond software v. 7.7.

**Figure 5:**
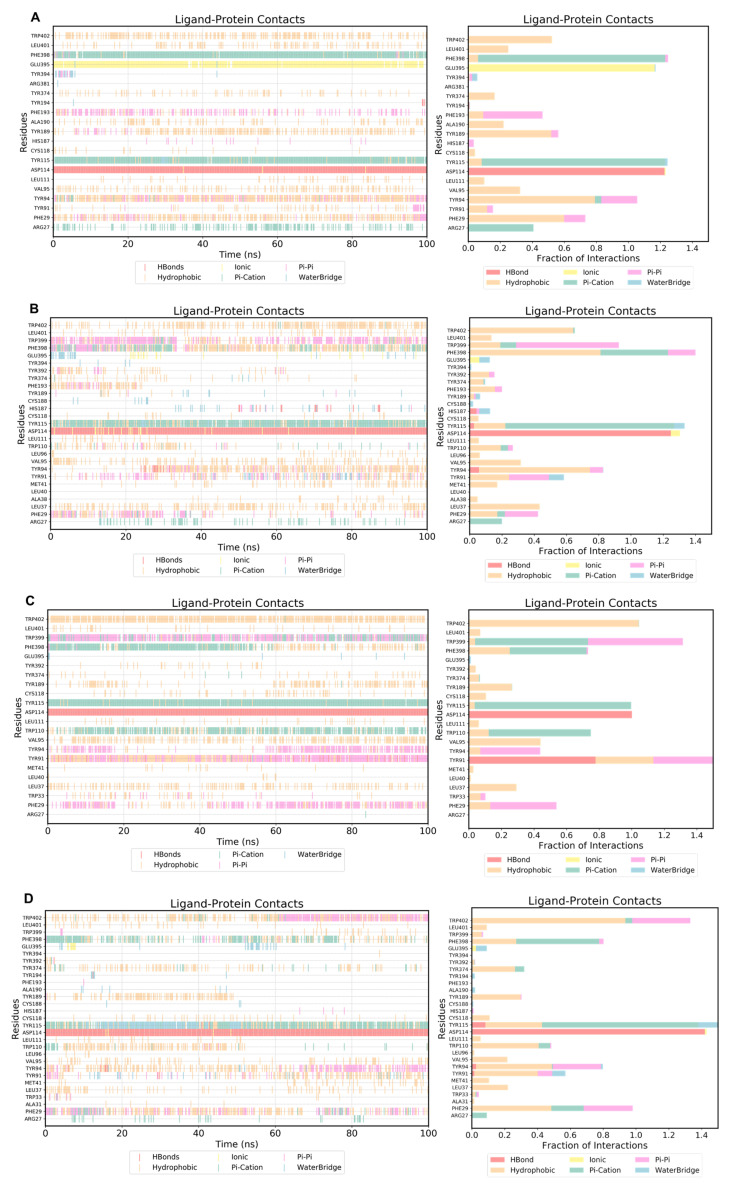
Ligand–protein contact plots. Plot (**A**) corresponds to ligand–protein system **6b**, plots (**B**) to **6e**, plots (**C**) to **6c**, and plots (**D**) to **6f**.

All complexes reached system stabilization with a root mean square deviation (RMSD) with a standard deviation of less than 1 Å (see Supplementary Materials). The most active compounds showed certain similarities in relation to binding site interactions, especially with the electrostatic interaction of Asp114 residue, regardless of alkyl chain extension. Compound **6b**, the smallest ligand with pronounced biological activity, showed persistence in electrostatic interactions with Glu395 residue and the piperidinium cation, which would explain its biological activity due to the accommodation of the cation in the innermost pocket of the binding site. In the case of compound **6e**, the interaction with Glu395 markedly decreased, and a bridge with a water molecule was added to enhance this interaction. Although this interaction is weak and occurs in the first part of the pathway, π-cation (Tyr315)- and π–π (Trp399)-type interactions were observed during molecular dynamics, leading to the accommodation of the central benzyl ring and terminal phosphine (Figure 5).

For less active compounds, such as **6c**, the interaction with Glu395 residue was almost absent, with π-π interactions dominating, responding to phosphine positioning. As the alkyl chain length increased, the phosphine moved further away from the binding site, exposing itself to the solvent and losing its affinity for the amino acids of the TM2 and TM7 domains. This explains why the ligand–protein contacts of compound **6f** were notably lower than those of the other synthesized ligands.

## 4. Conclusions

In this study, we have described the design, synthesis, as well as biological and in silico evaluation of six new multitarget directed ligands. The well-established 1-(3-phenoxypropyl) piperidine H_3_R pharmacophore was linked through alkyl chains of different lengths to a triphenylphosphonium cation, resulting in the first series of mitochondria targeting H_3_R ligands. High affinity for H_3_R could be maintained by our ligand design, comparable to the reference compound pitolisant. In addition, all derivatives showed weak to moderate antioxidant properties, which may nevertheless offer therapeutic benefits if the derivatives tend to accumulate in the mitochondria over time, regulating oxidative stress. Cytotoxicity studies on SH-SY5Y cells revealed that cell viability decreased with increasing alkyl chain length, while no cytotoxic effects at nanomolar ligand concentrations could be observed up to alkyl chain lengths of 5 carbon atoms. Molecular docking and molecular dynamics studies in the hH_3_R binding pocket indicated that compound **6e** creates significant interactions between the Asp114 residue and the protonated piperidine moiety and π–π stacking interactions between amino acid residues of Tyr39, Tyr94, Tyr189, and Phe193. Furthermore, the alkyltriphenylphosphonium moiety established hydrophobic and π-stacking interactions between the amino acid residues of Phe29, Arg27, Trp33, Leu37, Val95, and Trp399.

In view of our results, we described the first series of H_3_R ligands possibly targeting mitochondria as a starting point for a novel multitargeted approach as an alternative therapy for neurodegenerative disorders. Further modifications to improve antioxidant activity while maintaining a favorable balance between activity and toxicity are required in future studies.

## Figures and Tables

**Table 1:** Results of the biological evaluations of mitochondriotropic H_3_R ligands and references used.

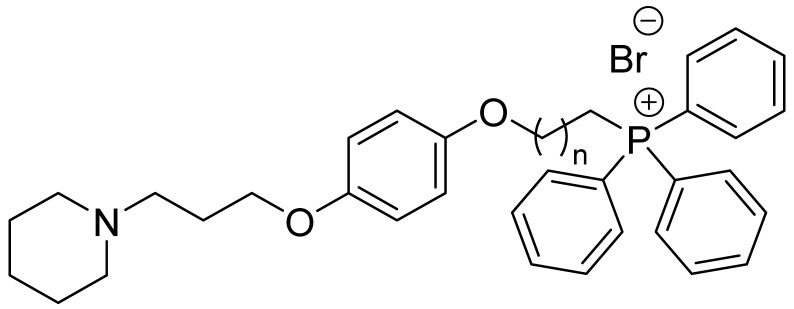				
Compound	*n*	hH_3_R K*_i_* [nM] ^a^	ORAC [TE] ^b^	LC_50_ [µM] ^c^
**6a**	3	32.0 [14.6; 70.0]	1.04 ± 0.03	40.0 [25.1; 63.6]
**6b**	4	17.6 [6.27; 49.4]	0.70 ± 0.01	21.8 [8.78; 54.2]
**6c**	5	31.5 [19.4; 51.3]	0.54 ± 0.03	11.2 [6.03; 20.8]
**6d**	7	20.4 [10.9; 38.4]	0.89 ± 0.16	2.69 [1.40; 5.16]
**6e**	8	14.1 [6.30; 31.7]	1.07 ± 0.06	0.91 [0.36; 2.28]
**6f**	9	67.4 [32.4; 140]	0.59 ± 0.06	2.61 [1.34; 5.08]
Pitolisant		13.2 [3.20; 54.1]	n.d.	n.d.
Caffeic acid		n.d.	3.08 ± 0.13	n.d.

^a^ [^3^H]*N*^α^-Methylhistamine displacement assay on HEK-293-hH_3_R membranes; K*_i_* [nM] with confidence interval at 95% of at least three independent experiments. ^b^ ORAC values expressed as mean trolox equivalents [TE] with standard error of the mean of at least three independent experiments. ^c^ LC_50_ [µM] determined after 24 h on SH-SY5Y cells with confidence interval at 95% of at least three independent experiments.

**Table 2:** IFD results for each of the synthesized ligands and their respective biological activities were obtained.

Compound	K*i* (nM)	pK*i*	IFD Score (kcal/mol)
**6a**	32.0	7.495	−11.343
**6b**	17.6	7.754	−12.624
**6c**	31.5	7.502	−12.204
**6d**	20.4	7.690	−13.339
**6e**	14.1	7.851	−14.378
**6f**	67.4	7.171	−10.433
Pitolisant	13.2		−15.324
PF-03654746	3.2 *		−15.321

* According to the reference [30].

## Data Availability

Data are contained within the article and Supplementary Materials.

## Supplementary Materials

The following supporting information can be downloaded at: https://www.mdpi.com/article/10.3390/antiox13111345/s1, Figure S1: Lateral view of the synthesized and docked ligands at the binding site. The compounds in yellow correspond to 6a (A), 6b (B), 6c (C), 6d (D), 6e (E), and 6f (F), while PF-03654746 is depicted in purple.; Figure S2: RMSD plots of complex 6b, 6e, 6c and 6f.; Figure S3: Frames of complex 6e and its conformations during the trajectory; Figure S4: RMSF plots of complex 6b, 6e, 6c and 6f.; Figure S5: RMSD plots of ligands during the trajectory 6b, 6e, 6c and 6f.; Figure S6: Radius of gyrate (rGyr) of the ligands 6b, 6e, 6c and 6f.; Figure S7: Solvent Accessible Surface Area (SASA) plots of 6b, 6e, 6c and 6f.; Figure S8: Contact interaction analysis of binding site residues with 6b. Contact scan during trajectory in the left plot and summary of interactions during trajectory time in the right plot.; Figure S9: Contact interaction analysis of binding site residues with 6c. Contact scan during trajectory in the left plot and summary of interactions during trajectory time in the right plot.; Figure S10: Contact interaction analysis of binding site residues with 6e. Contact scan during trajectory in the left plot and summary of interactions during trajectory time in the right plot.; Figure S11: Contact interaction analysis of binding site residues with 6f. Contact scan during trajectory in the left plot and summary of interactions during trajectory time in the right plot.; Figure S12: Estimated affinity energy during molecular dynamics per 5 ns for the following systems 6b, 6c, 6e y 6f.; Figure S13: hH3R competition binding curve of compounds 6a; Figure S14: hH3R competition binding curve of compounds 6b; Figure S15: hH3R competition binding curve of compounds 6c; Figure S16: hH3R competition binding curve of compounds 6d; Figure S17: hH3R competition binding curve of compounds 6e; Figure S18: hH3R competition binding curve of compounds 6f. References [37,38,39,40] are cited in the Supplementary Materials.
