# Supplementary material for: First Report on Cationic Triphenylphosphonium Compounds as Mitochondriotropic H3R Ligands with Antioxidant Properties
Source: Antioxidants (Basel). 2024 Nov 1;13(11):1345. doi: 10.3390/antiox13111345 (PMC11591188; doi:10.3390/antiox13111345)
Supplement: Supplementary file 1 [file antioxidants-13-01345-s001.zip › antioxidants-3263296-supplementary.pdf]

Supporting information

First Report on cationic triphenylphosphonium compounds as mitochondriotropic H_3R ligands with antioxidant properties

Tobias Werner [1], Tito Añazco [2], Paula Osses-Mendoza [2], Alejandro Castro-Álvarez [3], Cristian O. Salas [4], Raquel Bridi [5], Holger Stark [1,*], Christian Espinosa-Bustos [2,*]

[1]Institute of Pharmaceutical and Medicinal Chemistry, Heinrich Heine University Düsseldorf, Universitaetsstr. 1, 40225 Duesseldorf, Germany; **t.werner@hhu.de** (T.W.); stark@hhu.de (H.S.)

[2]Departamento de Farmacia, Facultad de Química y de Farmacia, Pontificia Universidad Católica de Chile, Santiago, 7820436, Chile; **taanazco@uc.cl** (T.A.); **paula.osses@uc.cl** (P.O.); ccespino@uc.cl (C.E-B.)

[3]Departamento de Ciencias Preclínicas, Facultad de Medicina, Universidad de La Frontera, Temuco 4780000, Chile; **Luis.castro@ufrontera.cl** (A.C-A.)

[4]Departamento de Química Orgánica, Facultad de Química y de Farmacia, Pontificia Universidad Católica de Chile, Santiago 7820436, Chile; **cosalas@uc.cl** (C.O.S.)

[5] Departamento de Química Farmacológica y Toxicológica, Facultad de Ciencias Químicas y Farmacéuticas, Universidad de Chile, Santiago 8380000, Chile; **raquelbridi@ciq.uchile.cl** (R.B.)

***Correspondence: ccespino@uc.cl (C.E-B.); stark@hhu.de (H.S.)**

^{1}H NMR compound 6a

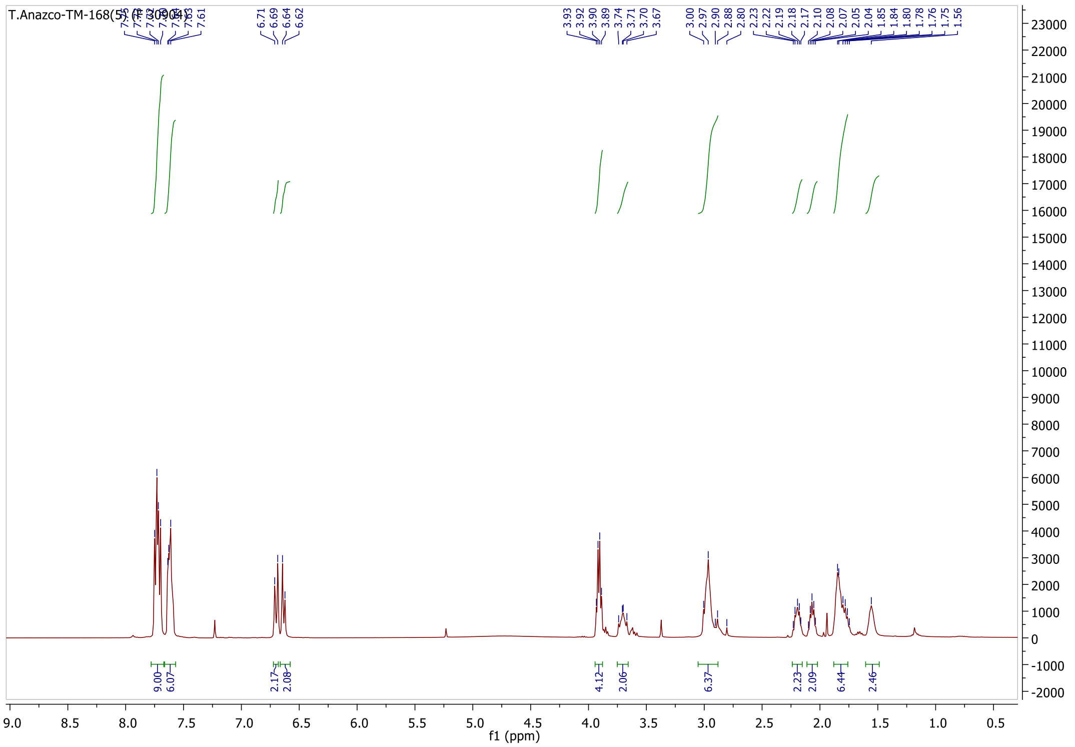

^{13}C NMR compound 6a

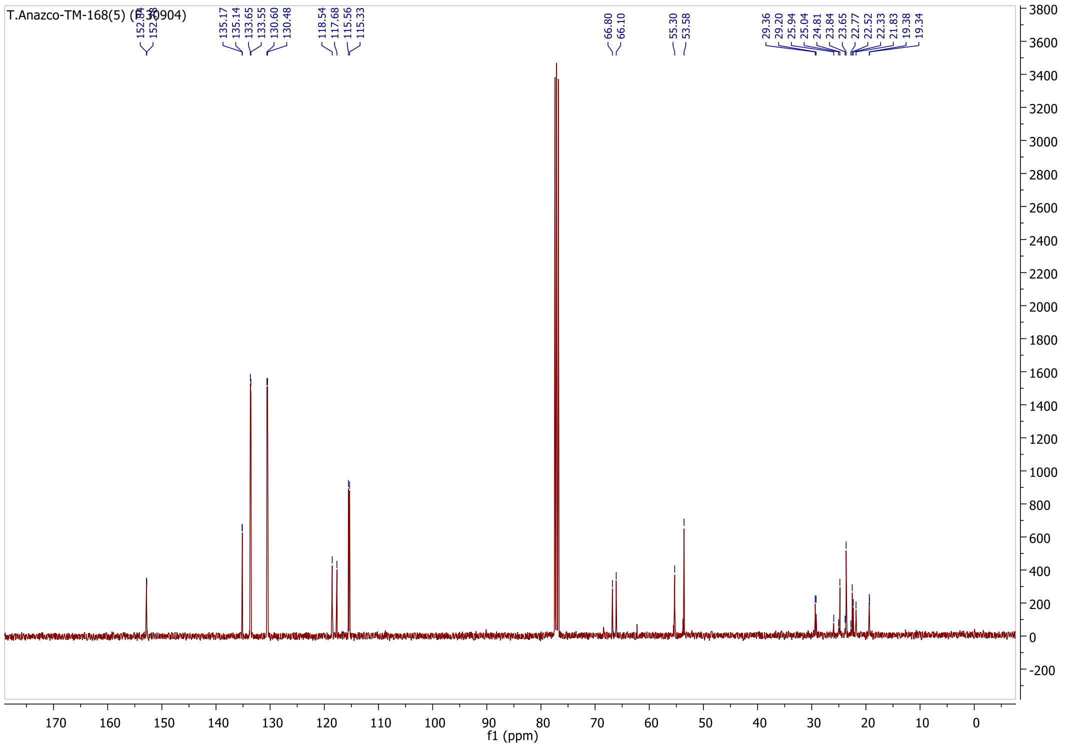

^{1}H NMR compound 6b

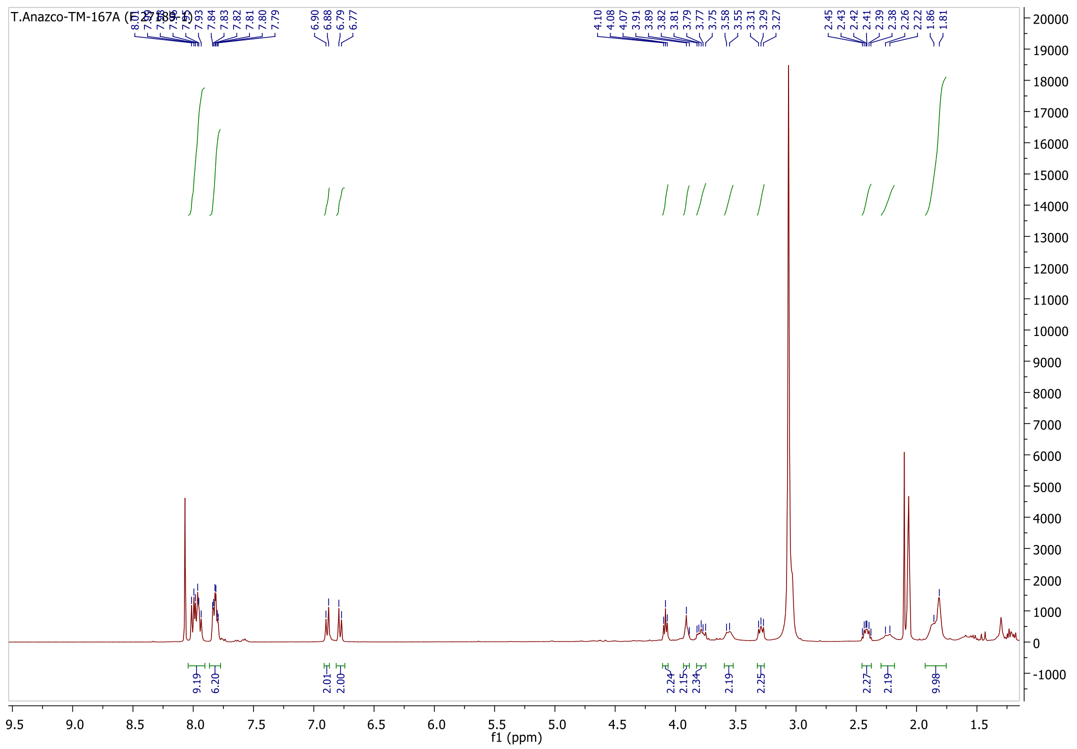

^{13}C NMR compound 6b

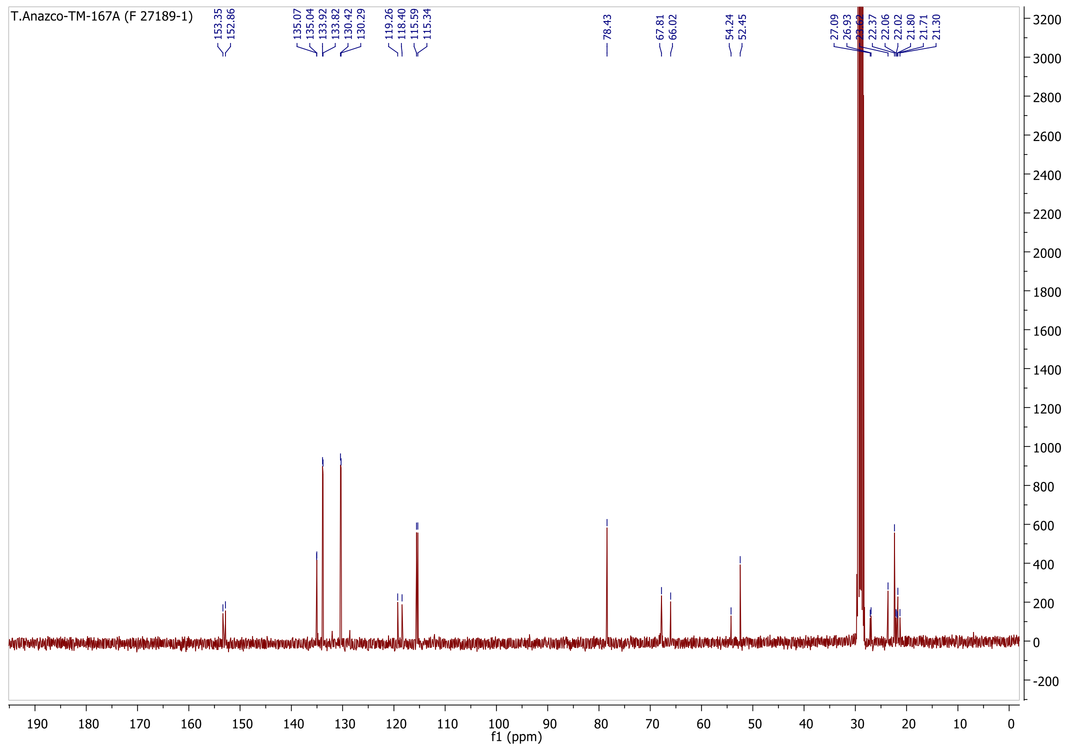

^{1}H NMR compound 6c

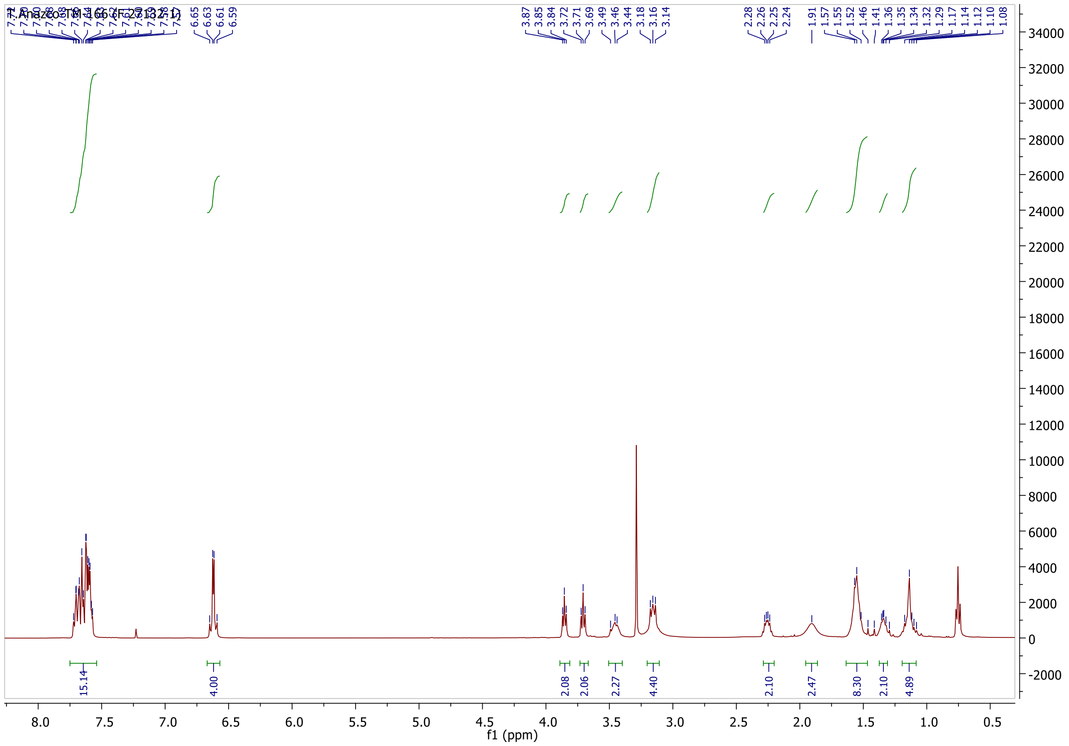

^{13}C NMR compound 6c

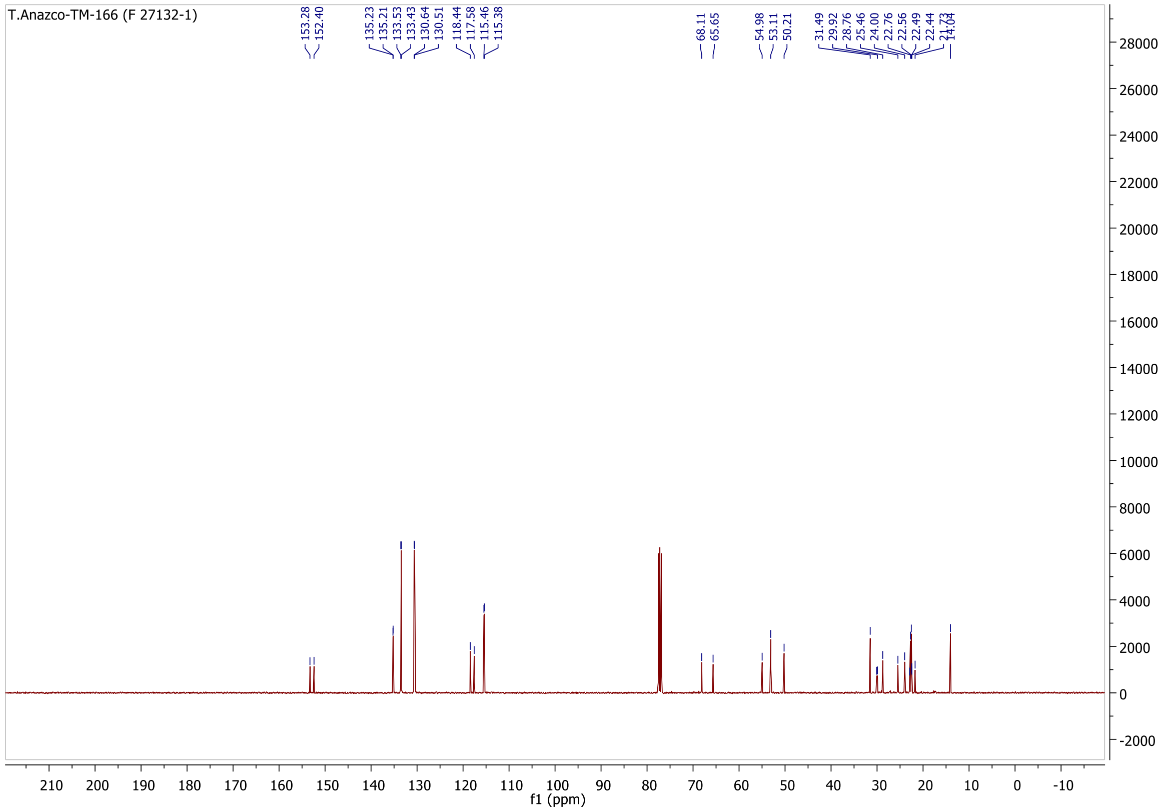

^{1}H NMR compound 6d

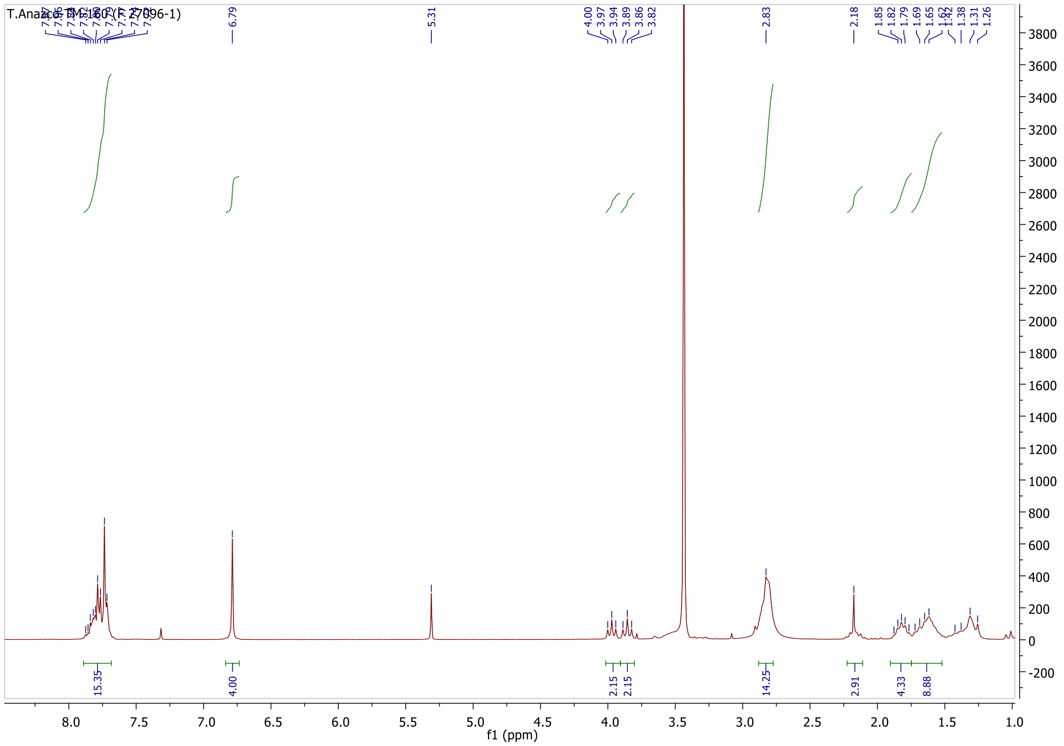

^{13}C NMR compound 6d

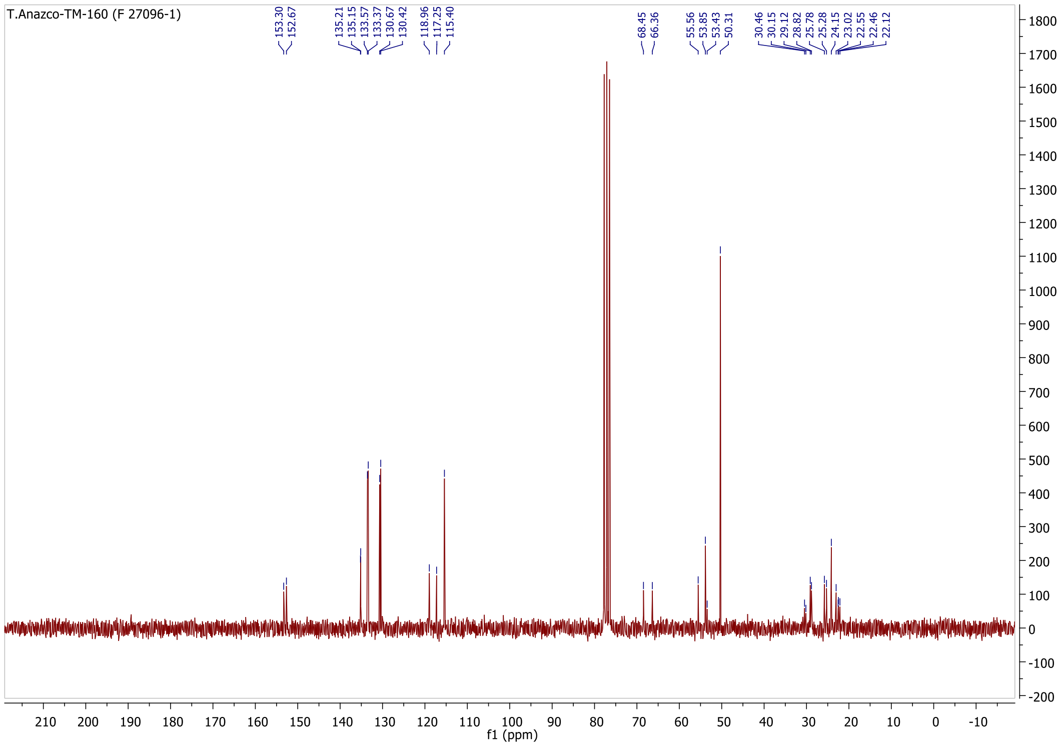

^{1}H NMR compound 6e

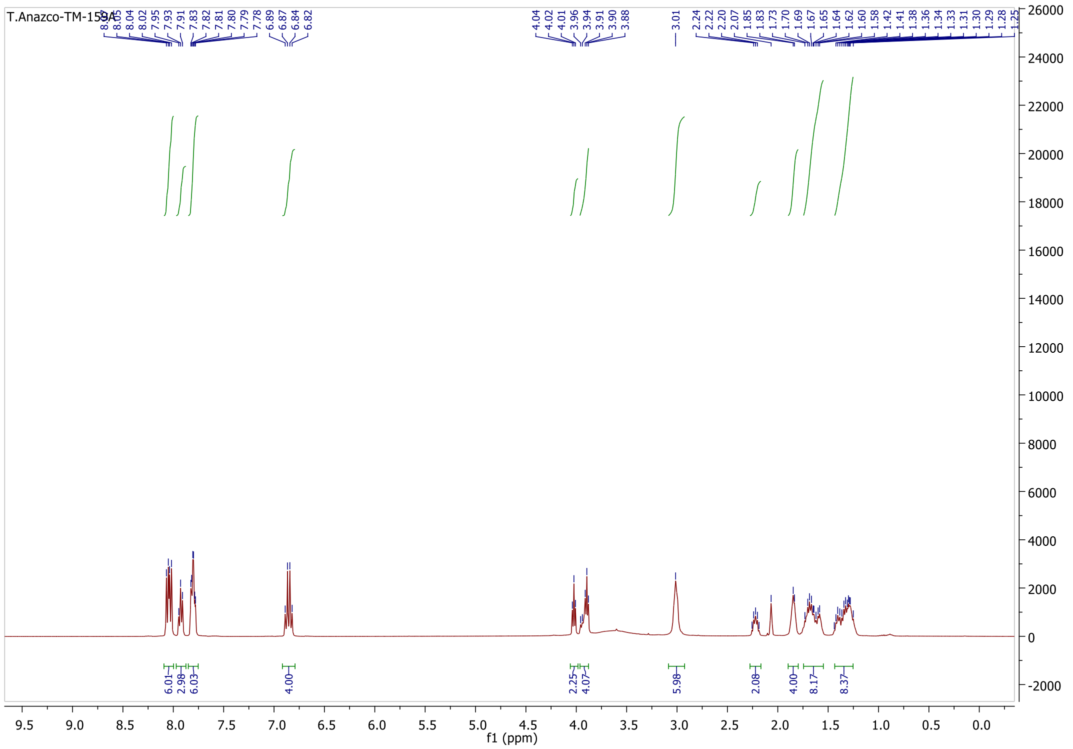

^{13}C NMR compound 6e

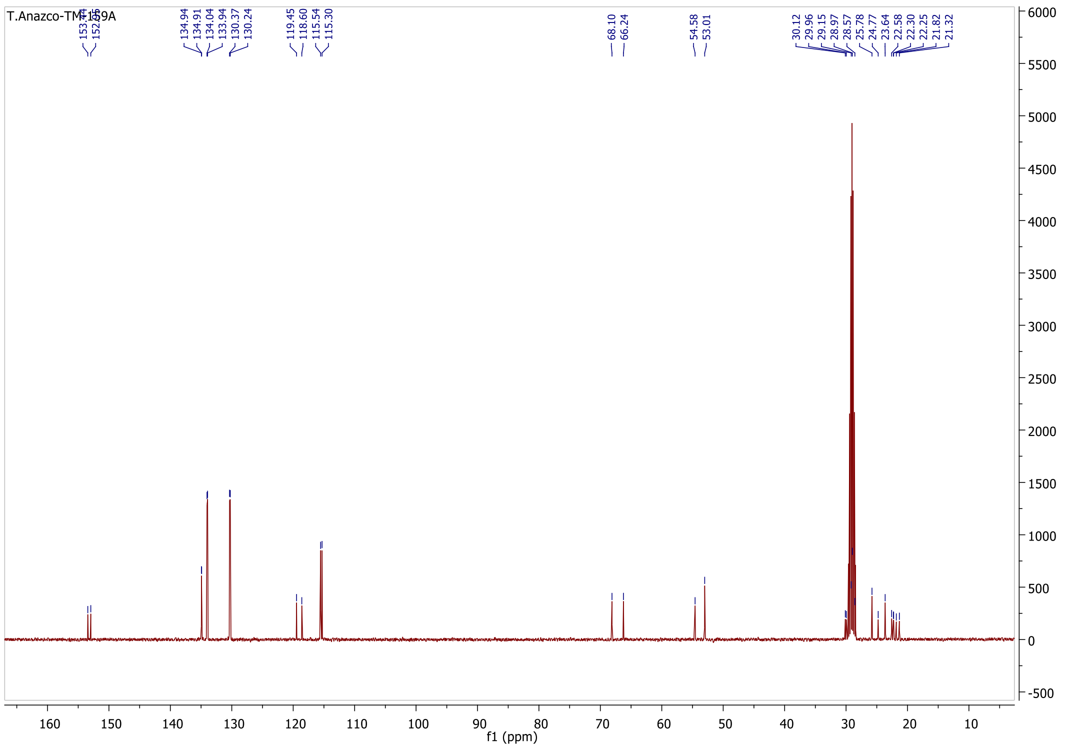

^{1}H NMR compound 6f

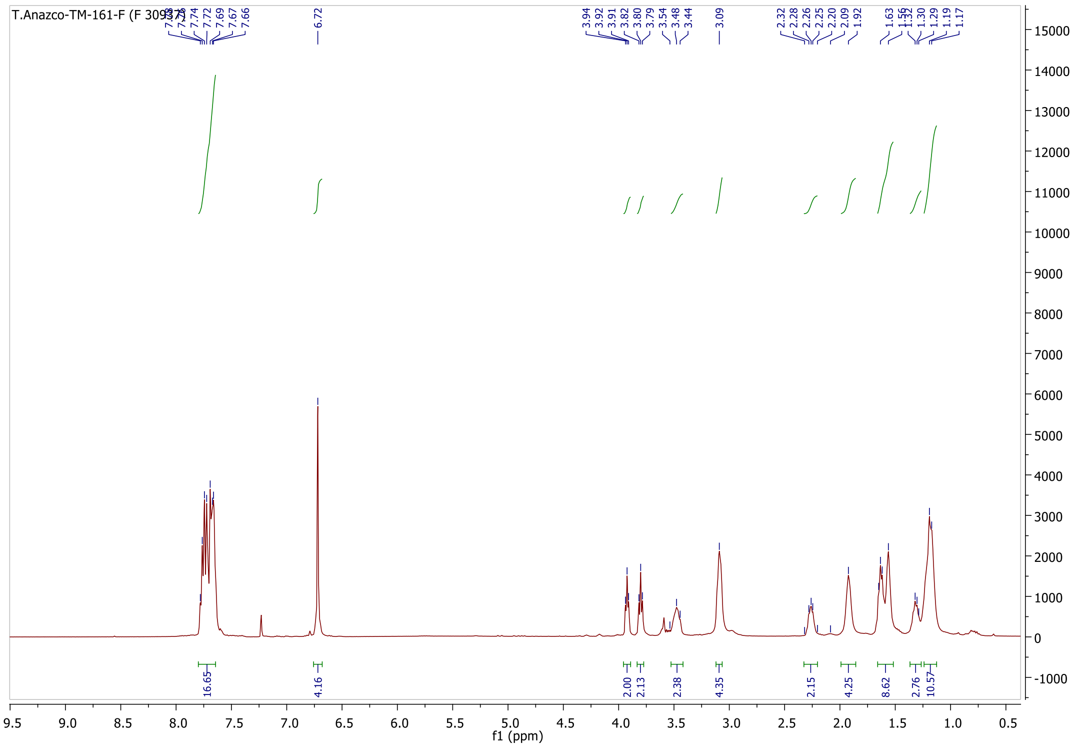

^{13}C NMR compound 6f

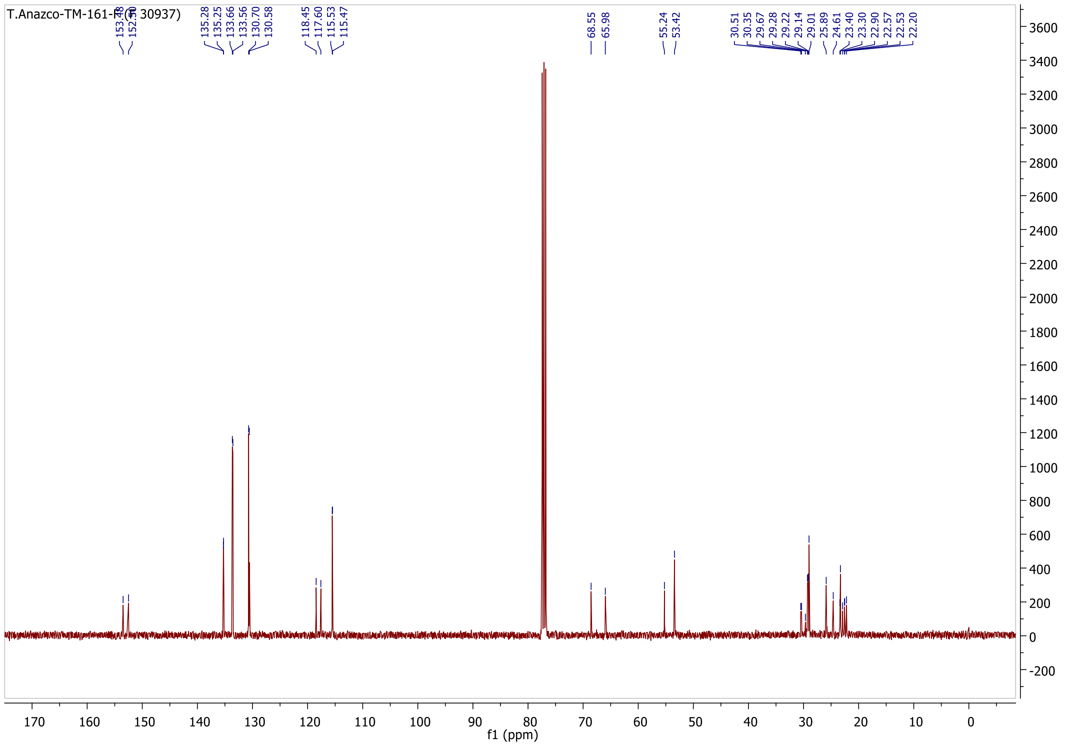

LC/HR-MS compound 6a

Compound Spectrum List Report

Analysis Info

Analysis Name	F:\Compact\2024\20022024\CE168_7_1_3807 — Blank_1_1_3801.d	Acquisition Date	2/20/2024 4:55:44 PM
Method	az 2bis 98 Reinheit ab 90 ab 200bis800nm pick up.m	Operator	Demo User
Sample Name	CE168	Instrument	compact 8255754.00000
Comment	Original analysis: 'CE168_7_1_3807.d',<br>Subtracted analysis: 'Blank_1_1_3801.d' (Xpose, 0.5s, 5),<br>Processed with: 'DataAnalysis 5.3.556',<br>Date: 2024-02-20T18:58:30		

Acquisition Parameter

Source Type	ESI	Ion Polarity	Positive	Set Nebulizer	1.8 Bar
Focus	Not active			Set Dry Heater	220 °C
Scan Begin	50 m/z	Set Capillary	4500 V	Set Dry Gas	9.0 l/min
Scan End	1300 m/z	Set End Plate Offset	-500 V	Set Divert Valve	Waste

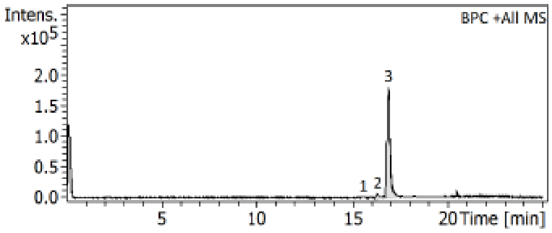

#	RT [min]	Area	S/N	Max. m/z	FWHM [min]	Area Frac. %
1	15.5	7305.4	15.6	308.2208	0.1	0.33
2	16.3	134243.3	142.0	218.2123	0.2	6.04
3	16.9	2079796.9	2140.3	276.6608	0.2	93.63

Cmpd 1, 15.5 min

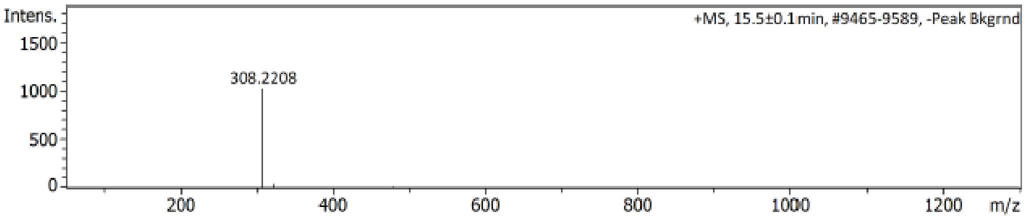

#	m/z	I %
1	308.2208	100.0
2	308.2677	15.8
3	308.2992	9.8
4	308.3372	11.1
5	308.3906	5.9
6	308.4392	4.2
7	322.2043	4.0
8	479.5074	1.3
9	648.5435	1.0
10	692.3985	1.0

Cmpd 2, 16.3 min

Compound Spectrum List Report

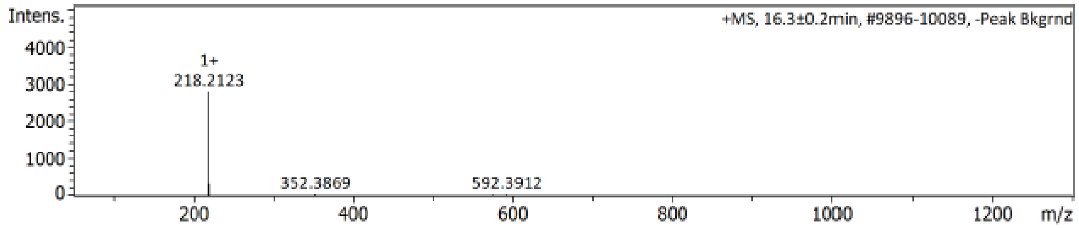

#	m/z	I %
1	218.2123	100.0
2	218.2760	2.2
3	218.3064	3.5
4	218.3302	2.4
5	218.3612	6.3
6	219.2162	12.3
7	219.2403	0.9
8	352.3869	2.0
9	575.3620	1.3
10	592.3912	2.0

Cmpd 3, 16.9 min

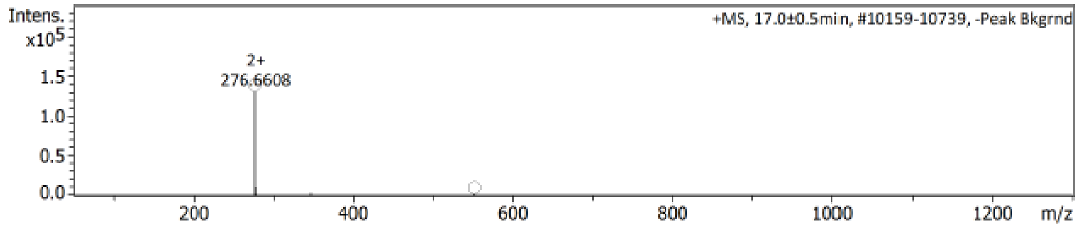

#	m/z	I %
1	276.6608	100.0
2	276.8047	0.7
3	276.8480	3.1
4	277.1622	40.8
5	277.3492	1.5
6	277.6637	8.4
7	278.1655	1.2
8	347.1558	2.2
9	552.3037	1.5
10	553.3112	0.7

#	z	Sum Formula	Adduct	Meas. m/z	err [mDa]	Ion Formula	m/z	err [ppm]	mSigma	# mSigma
1	2+	C36H42NO2P	M+H	276.6608	-5.9	C36H44NO2P	276.6549	-21.3	4.6	1
1	1+	C36H42NO2P	M+H	552.3037	-1.1	C36H43NO2P	552.3026	-2.0	32.4	1
2	2+	C33H46NO4P	M+H	276.6608	4.7	C33H48NO4P	276.6655	16.9	20.7	2
3	2+	C33H47NO2P2	M+H	276.6608	0.5	C33H49NO2P2	276.6614	2.0	21.7	3
4	2+	C31H42N3O4P	M+H	276.6608	-7.9	C31H44N3O4P	276.6529	-28.6	28.5	4
5	2+	C30H42N5O3P	M+H	276.6608	-2.3	C30H44N5O3P	276.6585	-8.3	30.9	5
6	2+	C29H42N7O2P	M+H	276.6608	3.3	C29H44N7O2P	276.6642	12.0	33.3	6
7	2+	C30H52NO2P3	M+H	276.6608	7.0	C30H54NO2P3	276.6678	25.3	38.6	7
8	2+	C29H46NO7P	M+H	276.6608	-3.0	C29H48NO7P	276.6579	-10.7	42.1	8
9	2+	C29H47NO5P2	M+H	276.6608	-7.1	C29H49NO5P2	276.6538	-25.6	43.0	9

LC/HR-MS compound 6b

Compound Spectrum List Report

Analysis Info

		Acquisition Date	2/20/2024 4:27:00 PM
Analysis Name	F:\Compact\2024\20022024\CE167_6_1_3806 — Blank_1_1_3801.d		
Method	az 2bis 98 Reinheit ab 90 ab 200bis800nm pick up.m	Operator	Demo User
Sample Name	CE167	Instrument	compact 8255754.00000
Comment	Original analysis: 'CE167_6_1_3806.d',<br>Subtracted analysis: 'Blank_1_1_3801.d' (Xpose, 0.5s, 5),<br>Processed with: 'DataAnalysis 5.3.556',<br>Date: 2024-02-20T18:49:36		

Acquisition Parameter

Source Type	ESI	Ion Polarity	Positive	Set Nebulizer	1.8 Bar
Focus	Not active			Set Dry Heater	220 °C
Scan Begin	50 m/z	Set Capillary	4500 V	Set Dry Gas	9.0 l/min
Scan End	1300 m/z	Set End Plate Offset	-500 V	Set Divert Valve	Waste

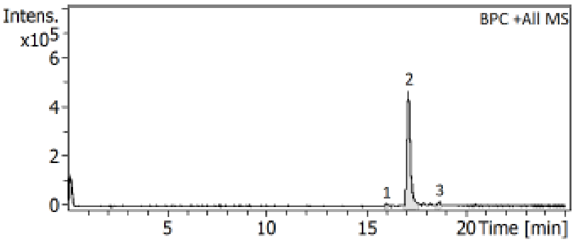

#	RT [min]	Area	S/N	Max. m/z	FWHM [min]	Area Frac. %
1	16.0	195081	235.3	322.2371	0.2	2.70
2	17.1	6546318	5894.5	283.6683	0.2	90.63
3	18.7	481656	407.2	372.7180	0.2	6.67

Cmpd 1, 16.0 min

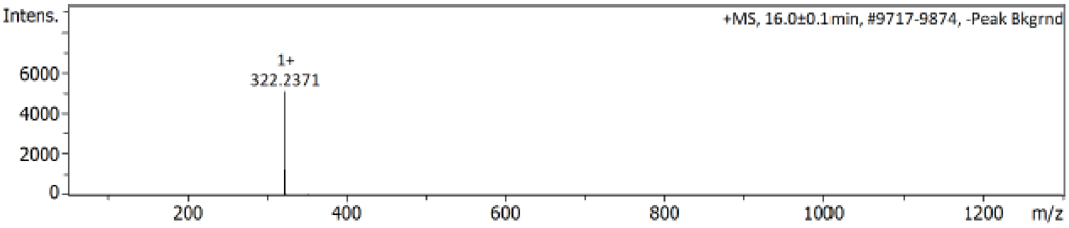

#	m/z	I %
1	322.2371	100.0
2	322.3283	2.3
3	322.3966	6.6
4	322.4358	3.3
5	322.4635	7.7
6	323.2404	25.5
7	323.2924	1.1
8	323.3517	1.8
9	323.4701	0.9
10	352.3854	1.1

Cmpd 2, 17.1 min

Compound Spectrum List Report

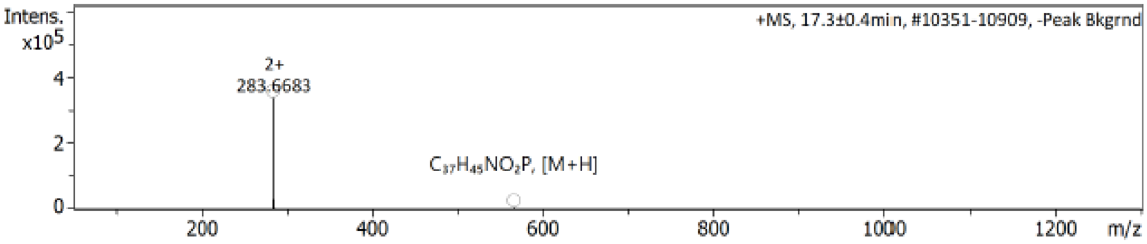

#	m/z	I %
1	283.5201	0.7
2	283.6683	100.0
3	283.8619	2.8
4	284.1696	41.4
5	284.3208	0.5
6	284.3633	1.4
7	284.6707	8.7
8	285.1724	1.1
9	566.3185	1.7
10	567.3221	0.7

#	z	Sum Formula	Adduct	Meas. m/z	err [mDa]	Ion Formula	m/z	err [ppm]	mSigma	# mSigma
1	2+	C37H44NO2P	M+H	283.6683	-5.6	C37H46NO2P	283.6628	-19.6	2.3	1
1	1+	C37H44NO2P	M+H	566.3185	-0.3	C37H45NO2P	566.3182	-0.5	7.0	1

Cmpd 3, 18.7 min

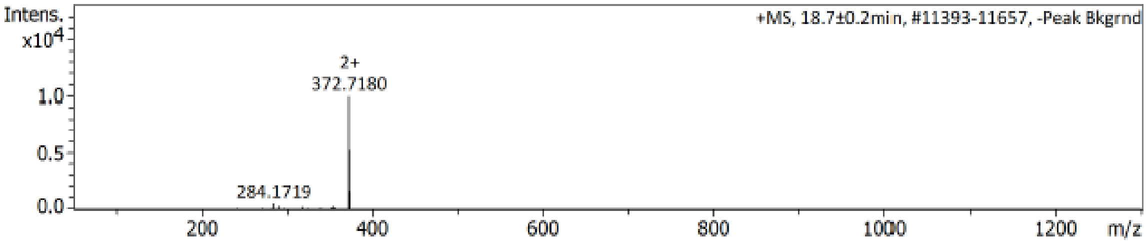

#	m/z	I %
1	283.6658	2.9
2	284.1719	5.2
3	290.6759	2.7
4	355.3198	2.9
5	372.7180	100.0
6	372.9347	2.4
7	372.9697	4.9
8	373.2203	53.9
9	373.7231	16.7
10	374.2263	3.2

LC/HR-MS compound 6c

Compound Spectrum List Report

Analysis Info

Analysis Name	F:\Compact\2024\20022024\CE166_5_1_3805 — Blank_1_1_3801.d	Acquisition Date	2/20/2024 3:58:15 PM
Method	az 2bis 98 Reinheit ab 90 ab 200bis800nm pick up.m	Operator	Demo User
Sample Name	CE166	Instrument	compact 8255754.00000
Comment	Original analysis: 'CE166_5_1_3805.d', Subtracted analysis: 'Blank_1_1_3801.d' (Xpose, 0.5s, 5), Processed with: 'DataAnalysis 5.3.556', Date: 2024-02-20T18:43:15		

Acquisition Parameter

Source Type	ESI	Ion Polarity	Positive	Set Nebulizer	1.8 Bar
Focus	Not active			Set Dry Heater	220 °C
Scan Begin	50 m/z	Set Capillary	4500 V	Set Dry Gas	9.0 l/min
Scan End	1300 m/z	Set End Plate Offset	-500 V	Set Divert Valve	Waste

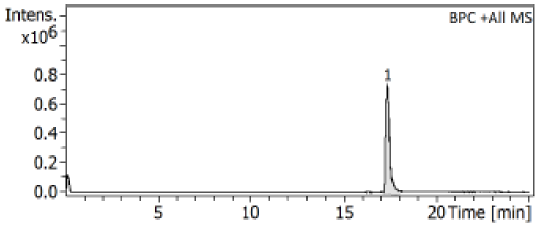

#	RT [min]	Area	S/N	Max. m/z	FWHM [min]	Area Frac. %
1	17.4	11749652	10065.5	290.6767	0.2	100.00

Cmpd 1, 17.4 min

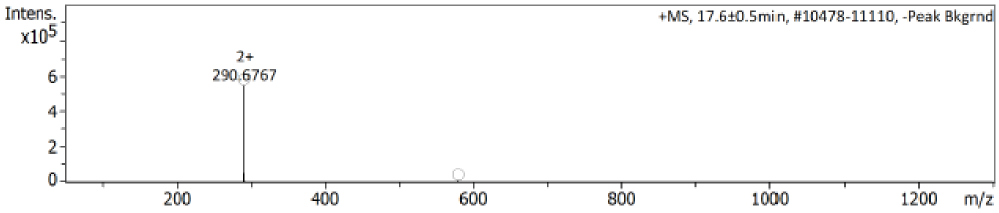

#	m/z	I %
1	290.5228	0.8
2	290.6767	100.0
3	290.8736	3.2
4	291.0153	0.5
5	291.1776	42.6
6	291.3759	1.4
7	291.6787	9.2
8	292.1803	1.3
9	580.3332	1.8
10	581.3366	0.8

#	z	Sum Formula	Adduct	Meas. m/z	err [mDa]	Ion Formula	m/z	err [ppm]	mSigma	# mSigma
1	2+	C38H46NO2P	M+H	290.6767	-6.1	C38H48NO2P	290.6706	-20.9	2.6	1
1	1+	C38H46NO2P	M+H	580.3332	0.7	C38H47NO2P	580.3339	1.3	5.1	1

LC/HR-MS compound 6d

Compound Spectrum List Report

Analysis Info

Analysis Name	F:\Compact\2024\20022024\CE160_3_1_3803 — Blank_1_1_3801.d	Acquisition Date	2/20/2024 3:00:48 PM
Method	az 2bis 98 Reinheit ab 90 ab 200bis800nm pick up.m	Operator	Demo User
Sample Name	CE160	Instrument	compact 8255754.00000
Comment	Original analysis: 'CE160_3_1_3803.d',<br>Subtracted analysis: 'Blank_1_1_3801.d' (Xpose, 0.5s, 5),<br>Processed with: 'DataAnalysis 5.3.556',<br>Date: 2024-02-20T18:30:56		

Acquisition Parameter

Source Type	ESI	Ion Polarity	Positive	Set Nebulizer	1.8 Bar
Focus	Not active			Set Dry Heater	220 °C
Scan Begin	50 m/z	Set Capillary	4500 V	Set Dry Gas	9.0 l/min
Scan End	1300 m/z	Set End Plate Offset	-500 V	Set Divert Valve	Waste

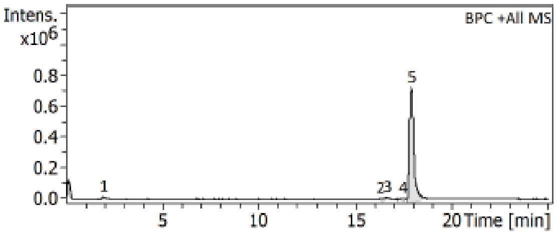

#	RT [min]	Area	S/N	Max. m/z	FWHM [min]	Area Frac. %
1	2.0	201145	221.9	132.9053	0.2	1.55
2	16.3	196443	263.7	218.2113	0.1	1.51
3	16.6	194125	225.2	258.2786	0.2	1.49
4	17.5	193400	228.4	364.2838	0.2	1.49
5	17.9	12203220	9399.5	304.6925	0.2	93.96

Cmpd 1, 2.0 min

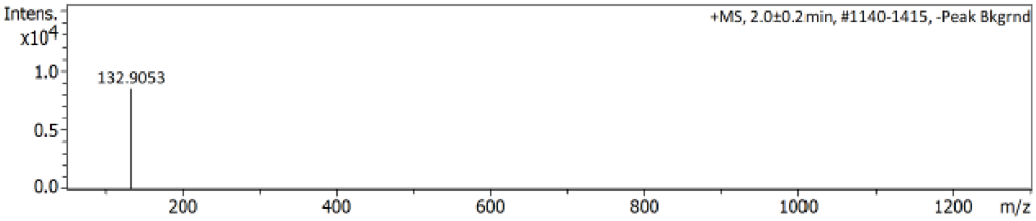

#	m/z	I %
1	132.9053	100.0
2	132.9765	0.7
3	132.9980	1.2
4	206.1382	0.5
5	206.1628	0.4
6	206.1802	0.4
7	236.8140	0.4
8	344.7347	0.8
9	346.7275	0.9
10	346.7773	0.5

Cmpd 2, 16.3 min

Compound Spectrum List Report

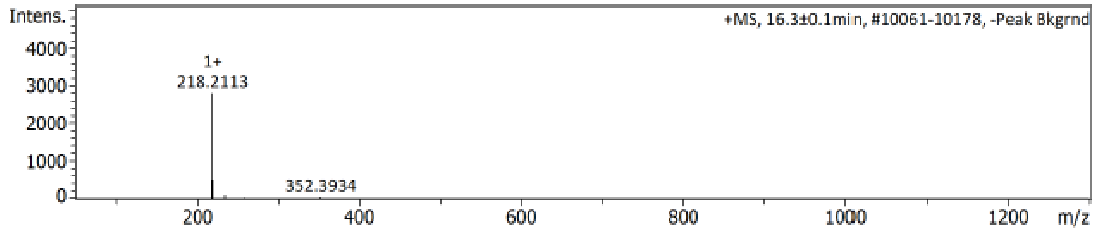

#	m/z	I %
1	218.2113	100.0
2	218.2906	4.2
3	218.3417	7.6
4	218.3714	4.2
5	219.2152	18.7
6	219.2490	3.5
7	219.2827	1.2
8	234.2061	3.4
9	258.3215	1.1
10	352.3934	2.1

Cmpd 3, 16.6 min

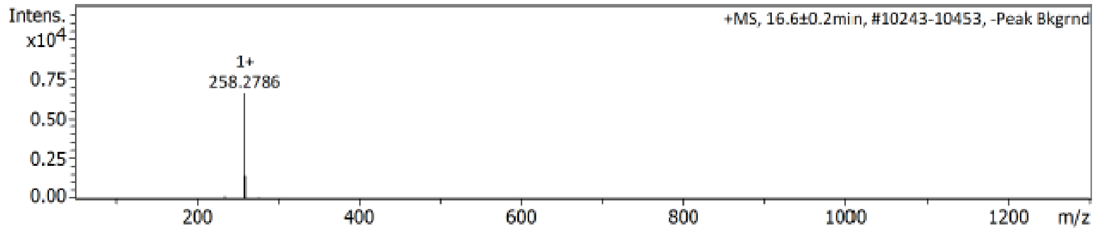

#	m/z	I %
1	234.2081	2.2
2	258.2786	100.0
3	258.3497	1.2
4	258.4308	4.1
5	258.4624	6.4
6	259.2817	22.7
7	259.3102	1.9
8	259.3556	1.4
9	259.4025	1.3
10	277.1117	1.2

Cmpd 4, 17.5 min

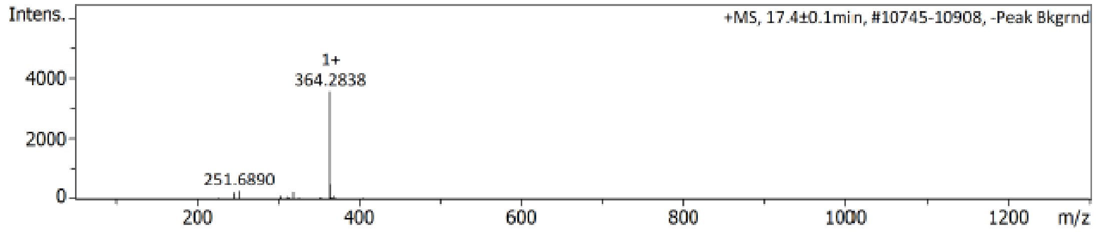

#	m/z	I %
1	246.2425	6.2
2	251.6890	7.7
3	251.7340	3.5

Compound Spectrum List Report

#	m/z	I %
4	319.7009	6.8
5	364.2838	100.0
6	364.3879	3.3
7	364.4357	7.8
8	364.4832	3.8
9	364.5341	8.4
10	365.2868	14.4

Cmpd 5, 17.9 min

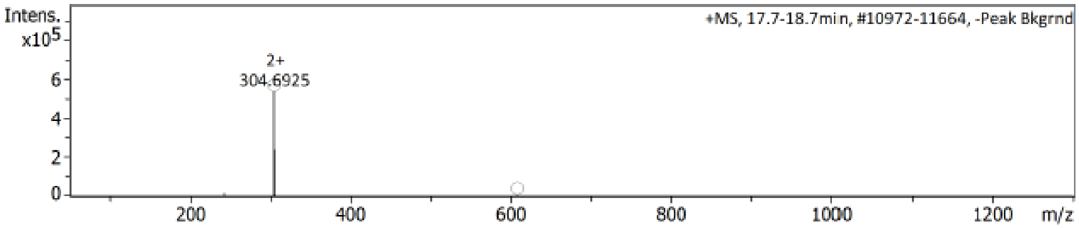

#	m/z	I %
1	242.2837	2.8
2	304.5291	0.8
3	304.6925	100.0
4	304.8984	3.5
5	305.1937	45.2
6	305.3980	1.9
7	305.6943	10.2
8	306.1960	1.6
9	608.3663	1.5
10	609.3690	0.7

#	z	Sum Formula	Adduct	Meas. m/z	err [mDa]	Ion Formula	m/z	err [ppm]	mSigma	# mSigma
1	2+	C40H50NO2P	M+H	304.6925	-6.3	C40H52NO2P	304.6862	-20.6	4.8	1
1	1+	C40H50NO2P	M+H	608.3663	-1.1	C40H51NO2P	608.3652	-1.8	7.8	1

LC/HR-MS compound 6e

Compound Spectrum List Report

Analysis Info

		Acquisition Date	2/20/2024 2:32:05 PM
Analysis Name	F:\Compact\2024\20022024\CE159_2_1_3802 — Blank_1_1_3801.d		
Method	az 2bis 98 Reinheit ab 90 ab 200bis800nm pick up.m	Operator	Demo User
Sample Name	CE159	Instrument	compact 8255754.00000
Comment	Original analysis: 'CE159_2_1_3802.d', Subtracted analysis: 'Blank_1_1_3801.d' (Xpose, 0.5s, 5), Processed with: 'DataAnalysis 5.3.556', Date: 2024-02-20T18:21:06		

Acquisition Parameter

Source Type	ESI	Ion Polarity	Positive	Set Nebulizer	1.8 Bar
Focus	Not active			Set Dry Heater	220 °C
Scan Begin	50 m/z	Set Capillary	4500 V	Set Dry Gas	9.0 l/min
Scan End	1300 m/z	Set End Plate Offset	-500 V	Set Divert Valve	Waste

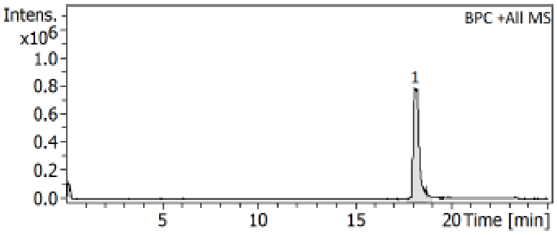

#	RT [min]	Area	S/N	Max. m/z	FWHM [min]	Area Frac. %
1	18.1	18916042	12892.9	311.7005	0.3	100.00

Cmpd 1, 18.1 min

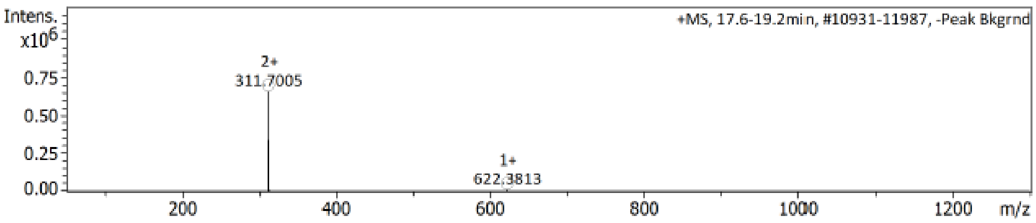

#	m/z	I %
1	311.5286	0.9
2	311.7005	100.0
3	311.9094	4.5
4	312.0277	0.6
5	312.2008	51.8
6	312.4121	2.0
7	312.7014	12.1
8	313.2030	1.9
9	622.3813	2.1
10	623.3850	0.9

#	z	Sum Formula	Adduct	Meas. m/z	err [mDa]	Ion Formula	m/z	err [ppm]	mSigma	# mSigma
1	2+	C41H52NO2P	M+H	311.7005	-6.5	C41H54NO2P	311.6941	-20.7	33.2	1
1	1+	C41H52NO2P	M+H	622.3813	-0.5	C41H53NO2P	622.3808	-0.8	7.5	1

LC/HR-MS compound 6f

Compound Spectrum List Report

Analysis Info

Analysis Name	F:\Compact\2024\20022024\CE161_4_1_3804 — Blank_1_1_3801.d	Acquisition Date	2/20/2024 3:29:31 PM
Method	az 2bis 98 Reinheit ab 90 ab 200bis800nm pick up.m	Operator	Demo User
Sample Name	CE161	Instrument	compact 8255754.00000
Comment	Original analysis: 'CE161_4_1_3804.d',<br>Subtracted analysis: 'Blank_1_1_3801.d' (Xpose, 0.5s, 5),<br>Processed with: 'DataAnalysis 5.3.556',<br>Date: 2024-02-20T18:37:07		

Acquisition Parameter

Source Type	ESI	Ion Polarity	Positive	Set Nebulizer	1.8 Bar
Focus	Not active			Set Dry Heater	220 °C
Scan Begin	50 m/z	Set Capillary	4500 V	Set Dry Gas	9.0 l/min
Scan End	1300 m/z	Set End Plate Offset	-500 V	Set Divert Valve	Waste

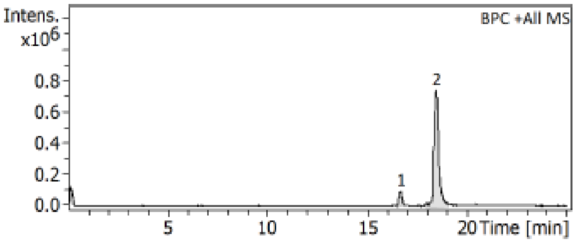

#	RT [min]	Area	S/N	Max. m/z	FWHM [min]	Area Frac. %
1	16.7	1155824	1532.1	277.1130	0.2	8.04
2	18.4	13214760	11335.7	318.7077	0.2	91.96

Cmpd 1, 16.7 min

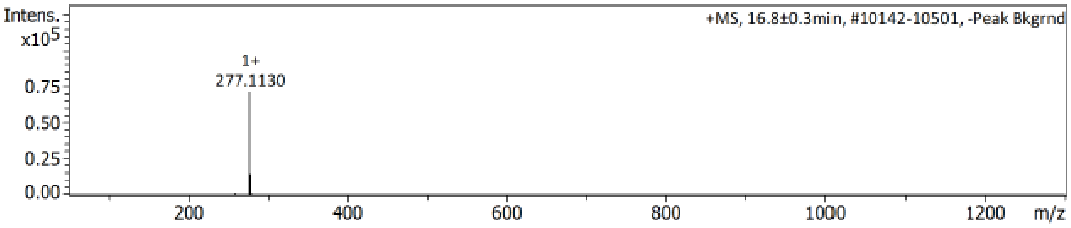

#	m/z	I %
1	258.2784	1.7
2	276.9710	0.5
3	277.1130	100.0
4	277.2639	1.1
5	277.3073	4.1
6	278.1163	21.2
7	278.2376	0.4
8	278.2797	0.6
9	278.3125	1.1
10	279.1193	2.1

Cmpd 2, 18.4 min

Compound Spectrum List Report

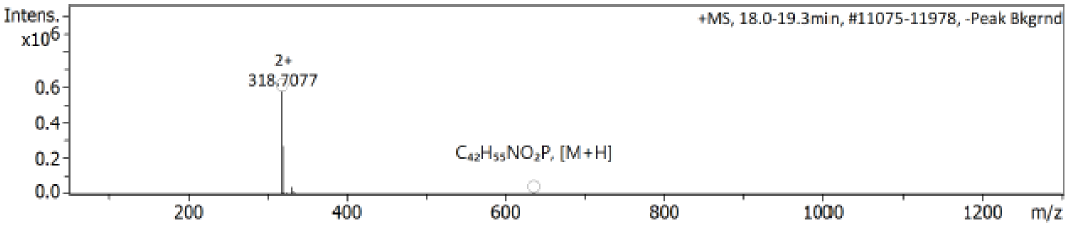

#	m/z	I %
1	318.7077	100.0
2	318.9222	3.9
3	319.2088	47.3
4	319.4247	1.9
5	319.7094	11.5
6	320.2110	1.8
7	325.7135	1.7
8	332.1731	7.0
9	332.6748	3.6
10	636.3964	1.6

#	z	Sum Formula	Adduct	Meas. m/z	err [mDa]	Ion Formula	m/z	err [ppm]	mSigma	# mSigma
1	2+	C42H54NO2P	M+H	318.7077	-5.9	C42H56NO2P	318.7019	-18.4	4.9	1
1	1+	C42H54NO2P	M+H	636.3964	0.1	C42H55NO2P	636.3965	0.2	4.7	1

S1. Induced-fit docking

Extension of the aliphatic chain provided the orientation of the phosphine according to its arrangement. Piperidine is located in the innermost region of the binding site and interacts with the Asp114 anion.

Phosphine is mostly composed of phenyl rings in the solvent-exposed region.

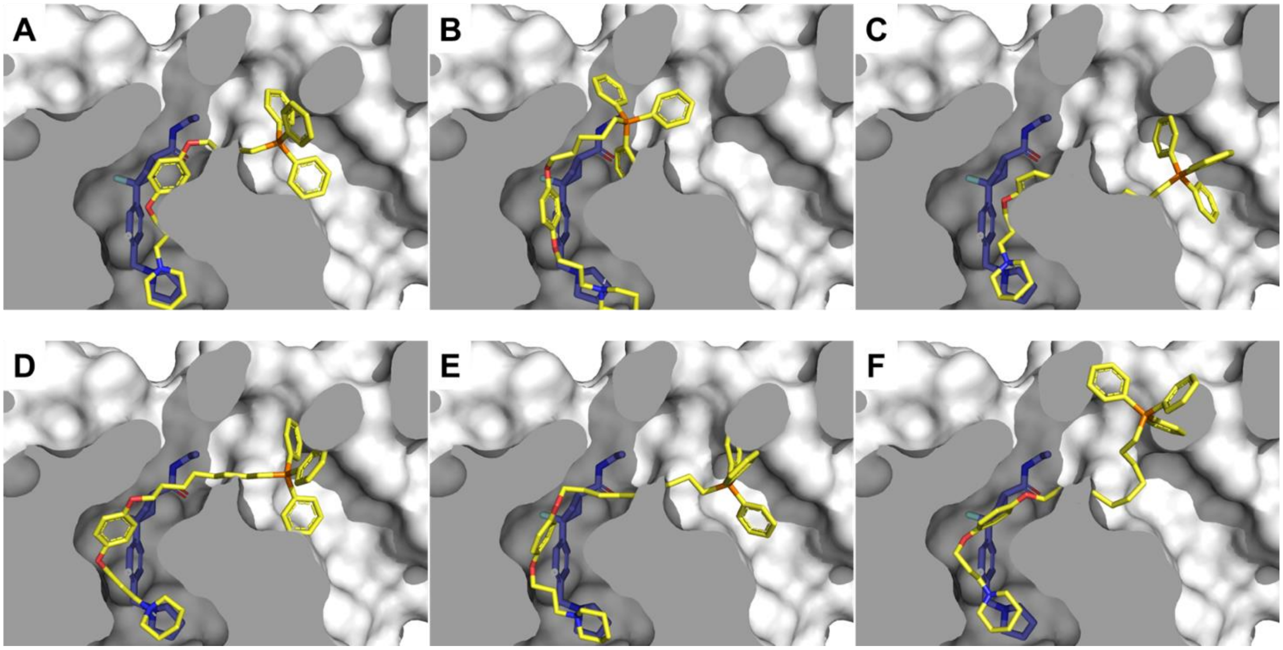

Figure S1. Lateral view of the synthesized and docked ligands at the binding site. The compounds in yellow correspond to **6a** (**A**), **6b** (**B**), **6c** (**C**), **6d** (**D**), **6e** (**E**), and **6f** (**F**), while PF-03654746 is depicted in purple.

S2. Molecular dynamics

The molecular dynamics equilibration process used at Desmond[1] follows a protocol that seeks to ensure stabilization of the system prior to performing the 100 ns molecular dynamics simulation (production). The details of each step are described below.

In the first step, a simulation using Brownian dynamics was performed on an NVT assembly (constant particle number, volume, and temperature), where the system temperature was set to 10 K. The initial objective of this step is to reduce the fluctuations of the system by applying small time steps with values of 0.001 ps and 0.003 ps. To avoid significant movements that could destabilize the simulation, constraints are imposed on the heavy atoms of the solute, typically proteins. The duration of this stage is 100 ps, during which the system is expected to reach an initial equilibrium state with minimal perturbations.

In the second stage, the system was subjected to a simulation in an NPT assembly (constant particle number, pressure, and temperature), where the temperature was increased to 50 K. At this point, a water barrier was introduced to allow the progressive relaxation of the solvent. The system pressure was kept constant while restraints were applied to both the membrane, restrained in the z-direction, and the protein to avoid abrupt displacements that could destabilize the system structure. This step allows for more precise control over the behavior of the water and membrane under specific temperature and pressure conditions.

The third stage continues to maintain the system temperature at 50 K but introduces an assembly NPgT (constant the number of particles, pressure, temperature, and pressure gradient), which allows finer tuning of the pressure conditions. The membrane remains constrained along the z-axis, and the protein remains constrained, ensuring that the overall structure is not affected by uncontrolled movements. The water barrier continues to be present, allowing the solvent to gradually adapt to the system conditions. This stage is crucial for preparing the system for controlled heating in subsequent steps, ensuring that the pressure and temperature equilibrium is stable.

In the fourth stage, the system was gradually heated from 100 to 310 K using an NPgT assembly. This step is essential for reaching physiological temperatures under controlled conditions. The water barrier remains active, and the restrictions on the membrane and proteins are gradually released, allowing the system to adapt smoothly to the temperature increase. This progressive release process is key to avoiding the introduction of structural stresses that could lead to collapse of the transmembrane system. Careful temperature elevation and the release of constraints ensure that the system reaches a thermodynamically favorable steady state.

The fifth stage marks the beginning of the production phase, in which all previously imposed constraints are removed. The system was simulated under an NVT assembly, ensuring that the temperature remained constant without the need to apply additional constraints to the system atoms. At this point, the system has reached a sufficient equilibrium state, and the removal of constraints allows molecular dynamics to more accurately reflect the natural behavior of the molecules in the simulated environment.

Finally, in the sixth step, the production phase was continued under NPT conditions without constraints. This step finalizes the equilibration process and starts the complete simulation, allowing the system to evolve freely according to the forces acting on atoms and molecules in the defined environment. This step is crucial for obtaining accurate and reproducible results, as the system is fully equilibrated and can reflect realistic molecular interactions without the influence of external constraints. From step 2 to step 6, equilibrations were performed for 1 ns per step to reach the optimal system to simulate physiological conditions.

A brief summary of the steps explained above is provided

1. simulate, Brownian Dynamics NVT, T = 10 K, small timesteps, and restraints on solute heavy atoms, 100ps
2. simulate, 50 K, H2O Barrier, Brownian NPT, membrane restrained in z, protein restrained
3. simulate, 50 K, H2O Barrier, NPgT, membrane restrained in z, protein restrained
4. simulate, NPgT, Heating from 100 -> 300 K, H2O Barrier and gradual release of restrain
5. simulate, NVT production remove all restraints
6. simulate, NVT production remove all restraints
7. simulate, production (MD 100 ns)

In these simulations, the ligand-protein complexes of the most active compounds (**6b** and **6e**) were evaluated and compared with those of the least active compounds (**6c** and **6f**).

The most notable difference in the RMSD values was observed for compound **6e**, which stabilized the system at approximately 20 ns. Subsequently, constant stabilization of protein conformations was maintained throughout the entire simulated trajectory.

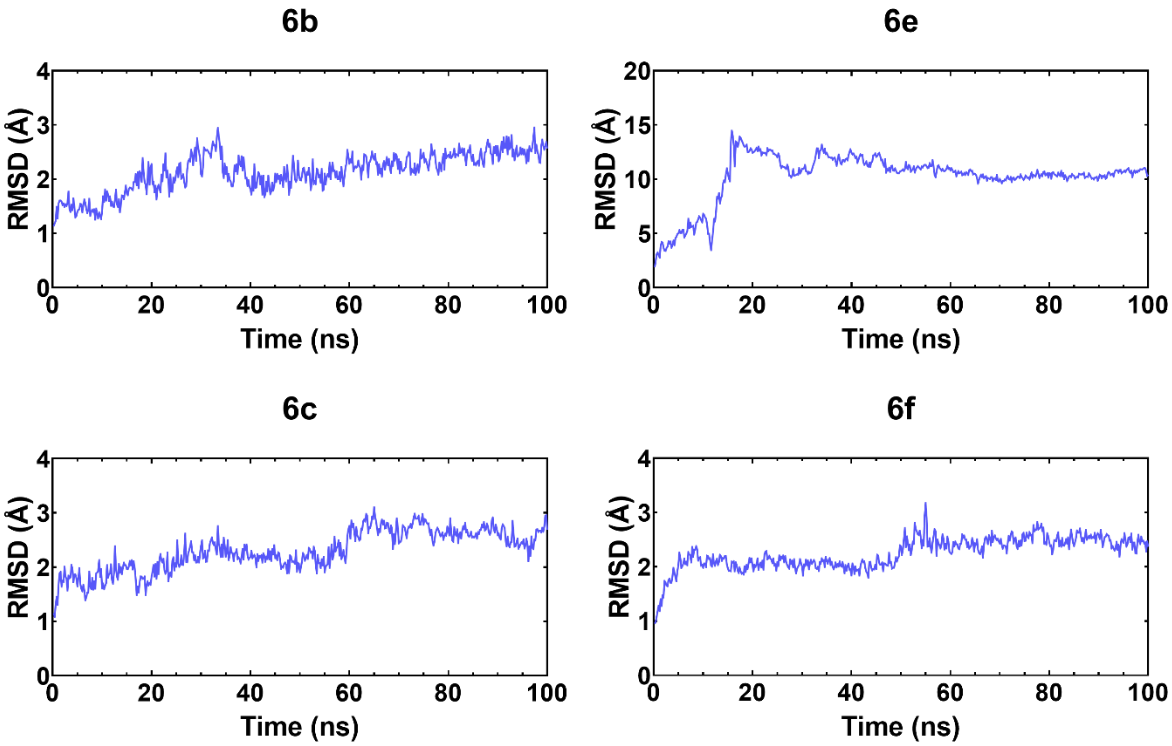

Figure S2. RMSD plots of complex **6b**, **6e**, **6c** and **6f**.

The alterations in the RMSD observed in the plot correspond to the conformational change of the aliphatic chain of compound **6e**, which adjusts the phosphine ligand between TM2 and TM7. This allows the positioning of piperidine in the innermost binding pocket, resulting in a more persistent interaction with Asp114.

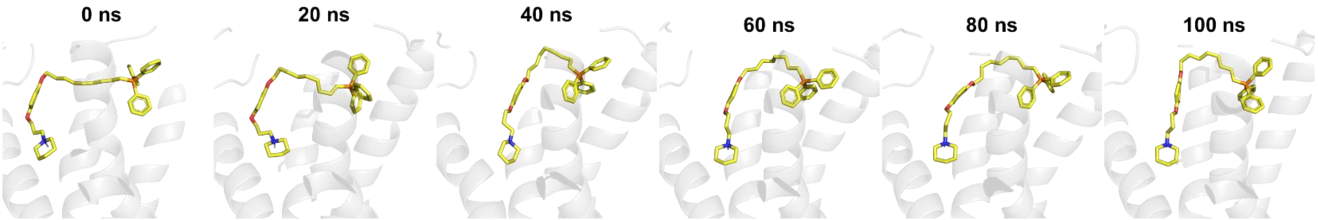

Figure S3. Frames of complex **6e** and its conformations during the trajectory.

This accommodation of the linker causes displacement of residues located in the region most exposed to the solvent of the binding site, generating conformational alterations in the protein. Such alterations could explain the fluctuations observed in the RMSD plot for complex **6e**.

Root Mean Square Fluctuation (RMSF) is a measure used in molecular dynamics to evaluate the fluctuation of the position of the atoms of the residues composing a protein at a given time. In the context of complex ligand-receptor analysis, RMSF allows the estimation of the stability and flexibility of the atoms in the receptor during simulation.

By analyzing the RMSF values of each system, it can be identified that ligands **6b**, **6c**, and **6f** present regions of the receptor with flexibility starting from amino acid 234, which consequently show better stabilization in the protein. However, compound **6e** had a higher RMSF in certain areas, which may indicate that these regions are susceptible to conformational changes, potentially affecting the interaction with the ligand and its influence on the H3R protein. below:

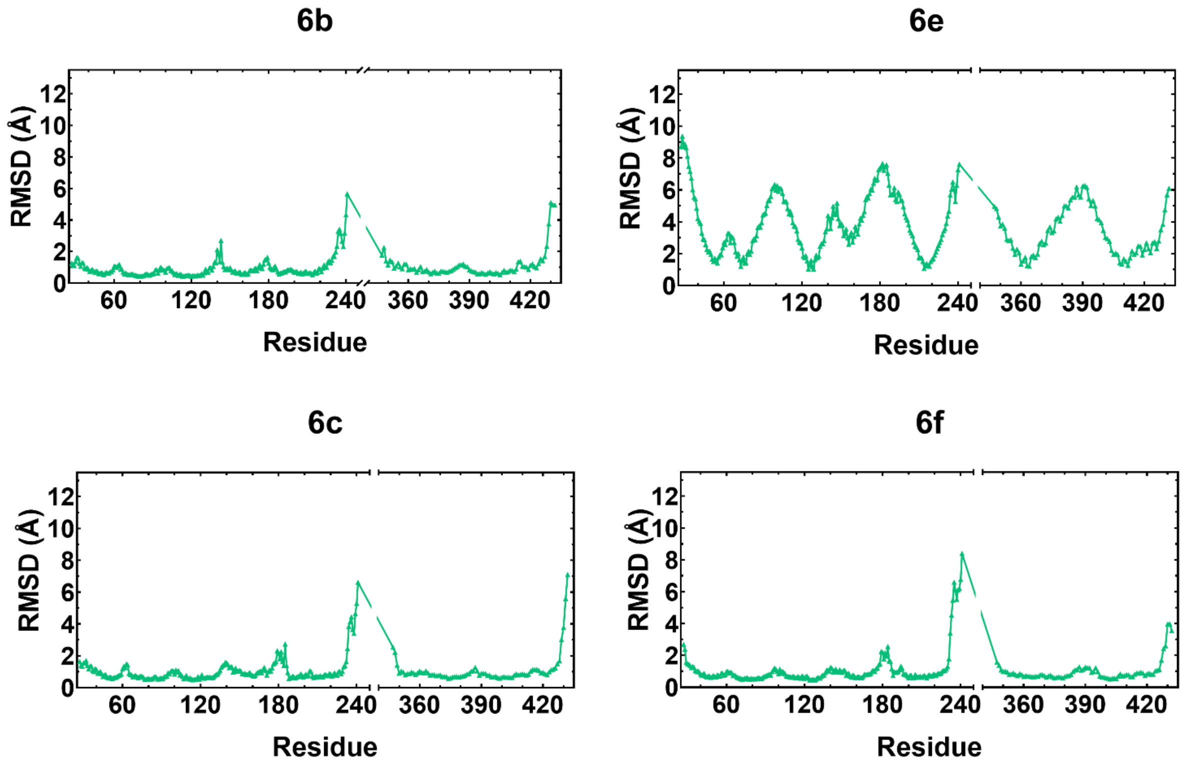

Figure S4. RMSF plots of complex **6b**, **6e**, **6c** and **6f**.

S2. Ligand properties during molecular dynamics

During molecular dynamics, we were able to extract three properties related to ligands **6b** and **6e** (more active) and **6c** and **6f** (less active). The RMSD of the ligands demonstrated the variability of the conformational changes that they underwent during the trajectory. Although compound **6e** presented high RMSD ranges in the protein (**Figure S3**), the RMSD of the ligand showed an increase in the position of its atoms up to 3 Å. However, compound **6f** reached RMSD values higher than 3 Å, which is due to the disposition of the phosphine towards the solvent-exposed region due to the extension of the side chain. In contrast, compound **6b** presents very low conformational variability because it is a smaller compound.

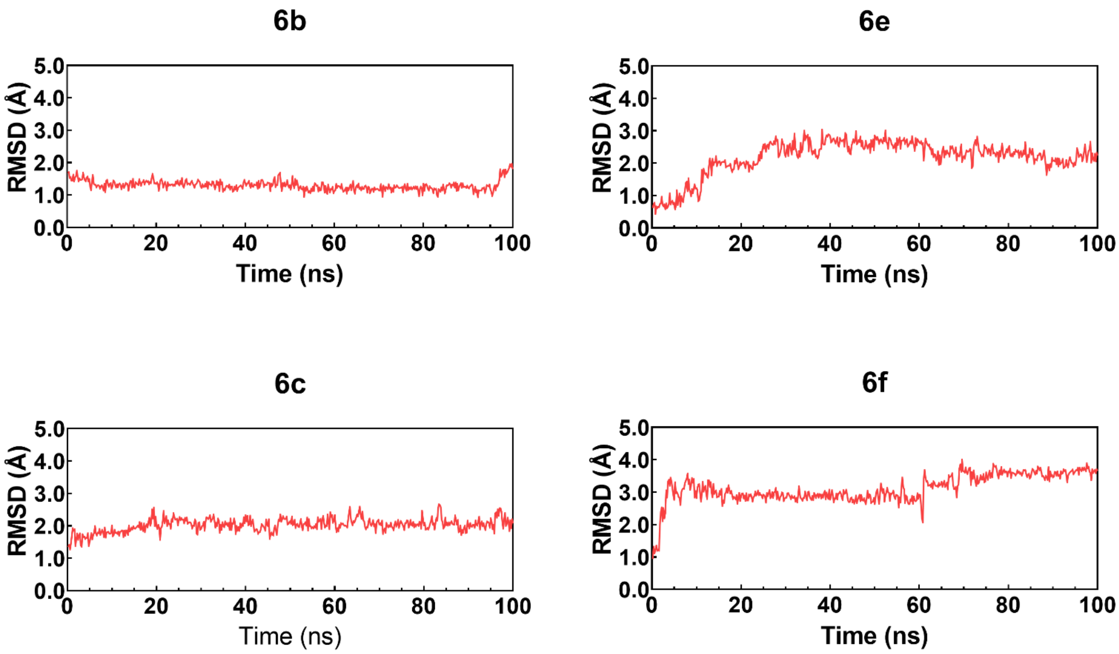

Figure S5. RMSD plots of ligands during the trajectory **6b**, **6e**, **6c** and **6f**.

In contrast, the radius of gyration reflects the distribution of the ligand atoms around its center of mass, which is useful for assessing the compaction and extension of the ligand at the binding site. A significant change in the radius of gyration can indicate alterations in the conformation of the ligand, which is crucial for understanding its behavior in a simulated biological environment.

In this case, compounds **6b** and **6c** showed less variability in their radius of gyration, indicating less conformational change during molecular dynamics. In contrast, compound 6e exhibited diverse conformational changes, reaching compactness at approximately 20 ns and extending slightly after 30 ns (**Figure S3**). Compound **6f**, on the other hand, starts with a more extended conformation (greater than 8 Å), and approximately 50 ns begins to compact, repositioning the phosphine ligand in the extracellular space.

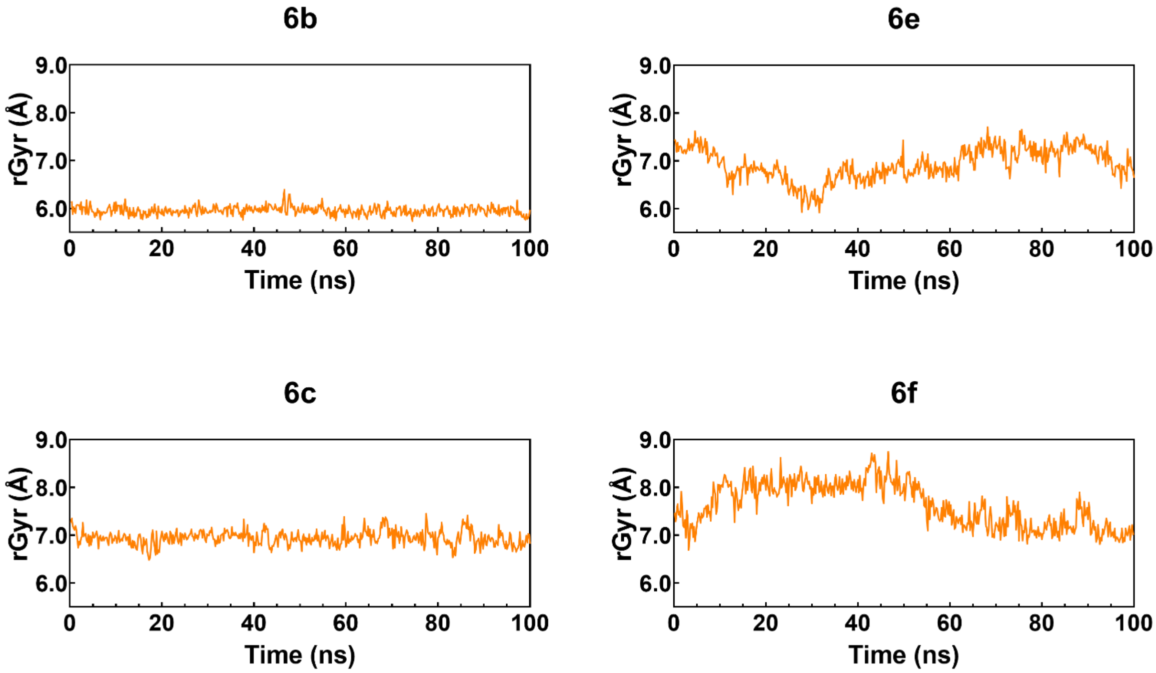

Figure S6. Radius of gyrate (rGyr) of the ligands **6b**, **6e**, **6c** and **6f**.

Finally, the Solvent-Accessible Surface Area (SASA) of the ligand quantifies the surface area of the ligand that is accessible to the solvent, providing information regarding its exposure and possible interactions with the aqueous medium. This parameter is particularly relevant for assessing the solubility and interaction of the ligand with its environment, in this case, the H3R receptor binding site. Compound **6b** showed conservative exposure resulting from the extension of piperidine to phosphine. Despite the various changes observed in compound **6e**, its solvent exposure was similar to that of compound **6c**, thus exhibiting less disposition to the extracellular space. In contrast, compound **6f** showed a remarkable interaction of its surface with the solvent, reaching values above 300 Å^2, which corresponds to the phosphine of the synthesized ligand.

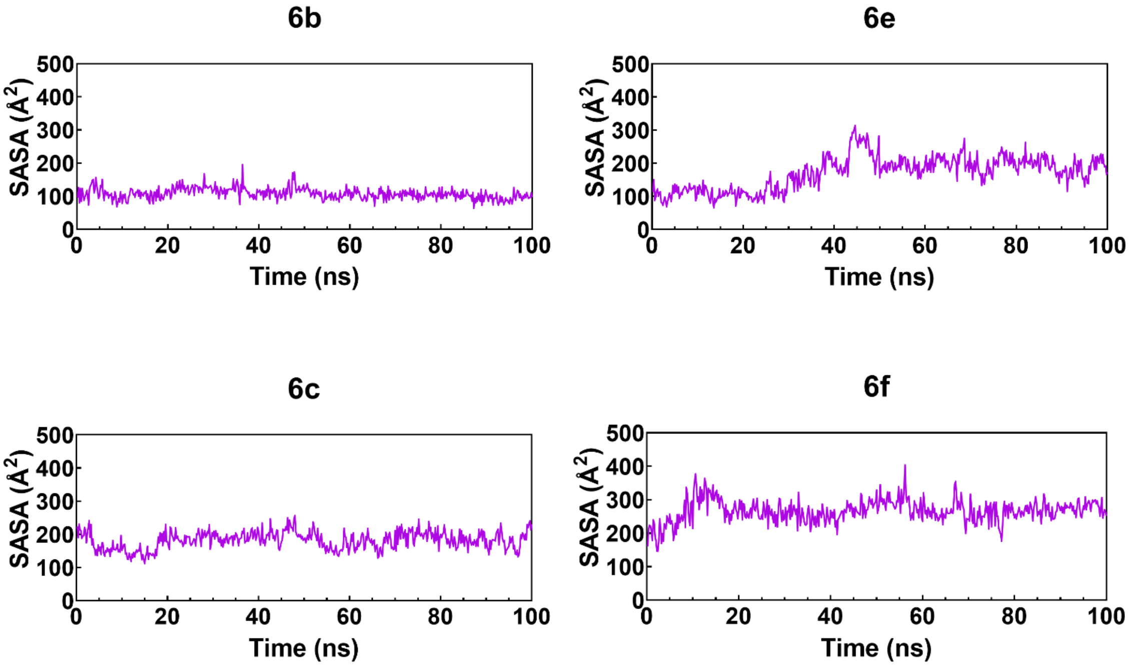

Figure S7. Solvent Accessible Surface Area (SASA) plots of **6b**, **6e**, **6c** and **6f**.

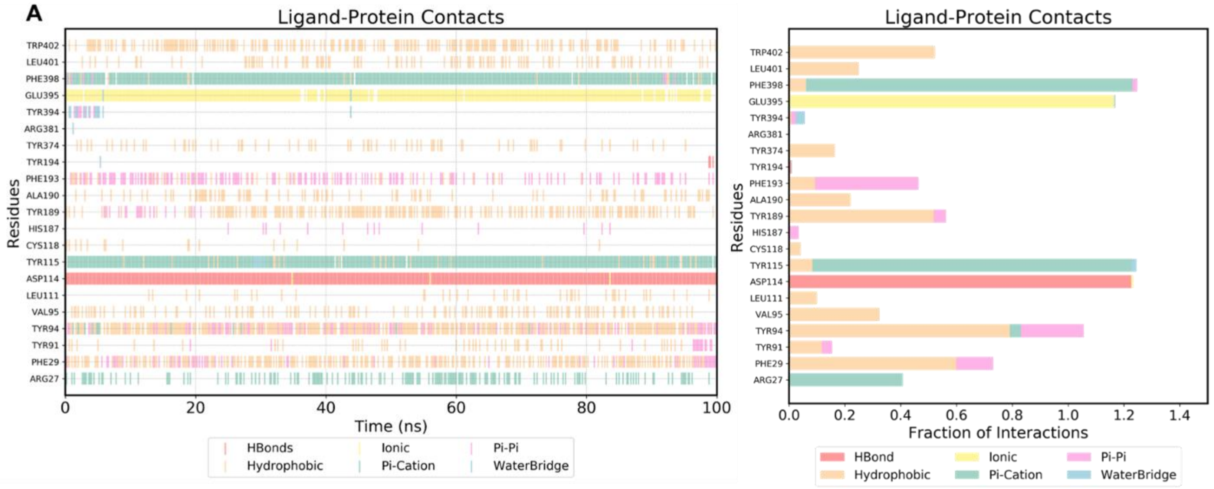

Figure S8. Contact interaction analysis of binding site residues with **6b**. Contact scan during trajectory in the left plot and summary of interactions during trajectory time in the right plot.

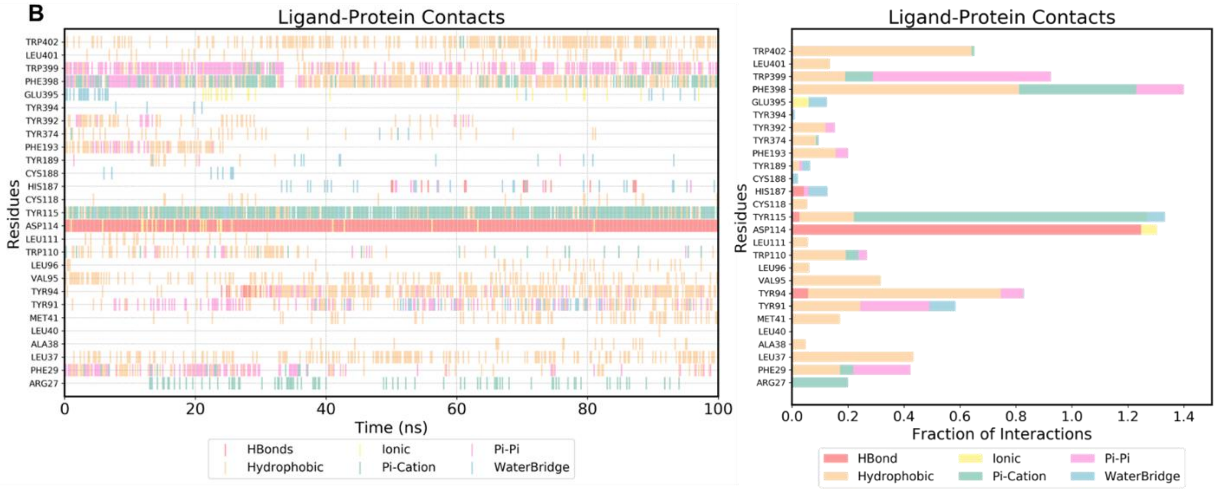

Figure S9. Contact interaction analysis of binding site residues with **6c**. Contact scan during trajectory in the left plot and summary of interactions during trajectory time in the right plot.

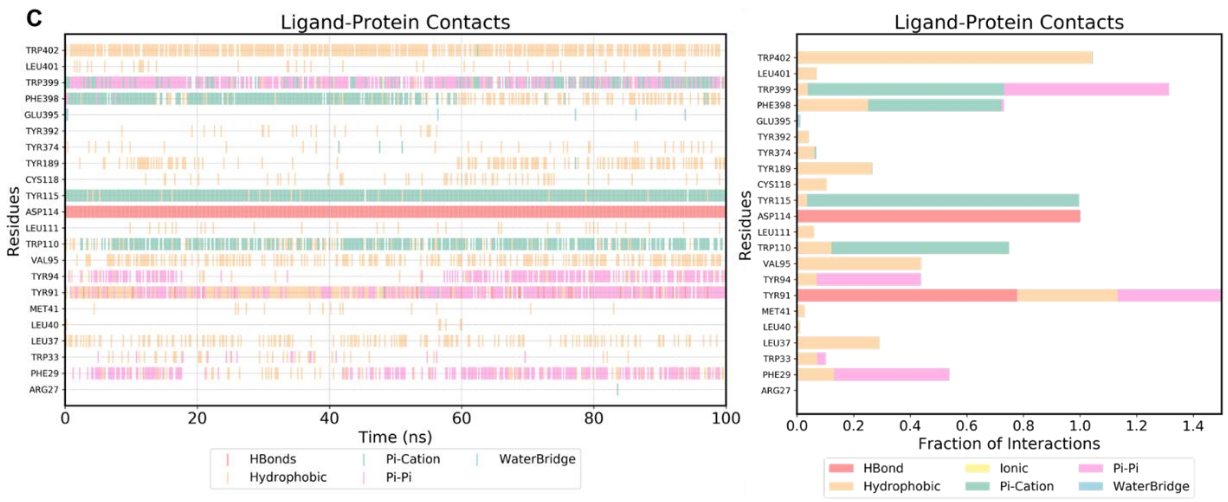

Figure S10. Contact interaction analysis of binding site residues with **6e**. Contact scan during trajectory in the left plot and summary of interactions during trajectory time in the right plot.

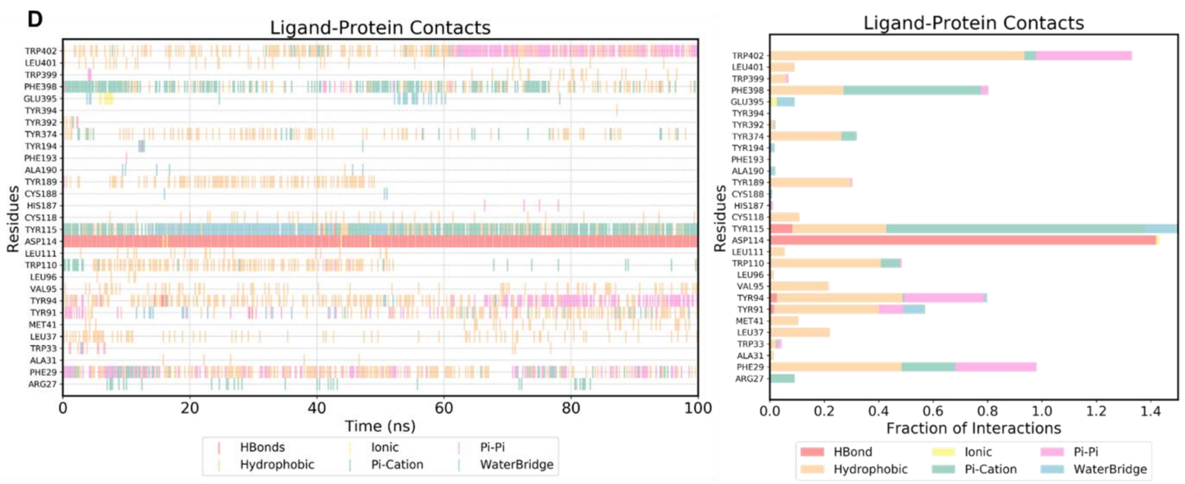

Figure S11. Contact interaction analysis of binding site residues with **6f**. Contact scan during trajectory in the left plot and summary of interactions during trajectory time in the right plot.

S3. MM-GBSA analysis

From the trajectories obtained for each system, binding energy (ΔG) estimation calculations were performed using the MM-GBSA method [2,3] with the Prime program [4]. For this purpose, 20 frames of each trajectory were analyzed. The averages of the resulting affinity energies were -63.69 kcal/mol for compound **6b**, -74.97 kcal/mol for compound **6e**, -45.72 kcal/mol for compound **6c**, and -55.67 kcal/mol for compound **6f**, as shown in Figure S8. In the case of the system with compound 6b, a decrease in energy was observed in the first frame, maintaining a constant interaction of approximately -50 kcal/mol starting at 40 ns. In contrast, the system with compound 6e showed an affinity higher than -70 kcal/mol starting at 10 ns and remained stable during the entire trajectory. In contrast, compounds 6c and 6f exhibited a clear decrease in the interaction with the binding site along the trajectory, reaching values below -60 kcal/mol during the molecular dynamics' simulation, which can be explained by the extension of the aliphatic chain and the interaction of the phosphine with the amino acids of TM2 and TM7.

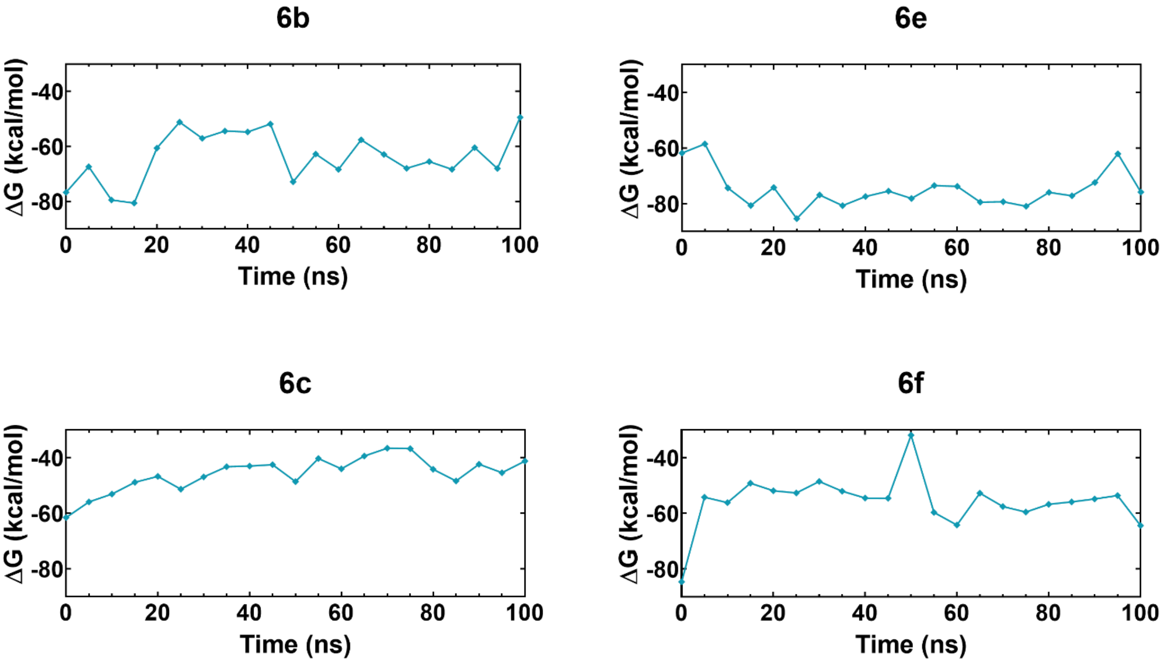

Figure S12. Energía de afinidad estimada durante la dinámica molecular por cada 5 ns para los sistemas **6b**, **6c**, **6e** y **6f**.

S4. hH_3R Radioligand Displacement Assay

Radioligand displacement assays were performed on the membrane fractions of HEK-293 cells stably expressing hH_3R. Cultivation of hH_3R-HEK-293 cells and membrane preparation was performed as described previously [5]. $[^3H]N^{\alpha}$-Methylhistamine was used as the radiolabeled probe at a final concentration of 2 nM (Kd = 3.08 nM).K_i values were calculated with Cheng-Prusoff equation and -log(Ki) was used to calculate mean values and 95% confidence intervals.

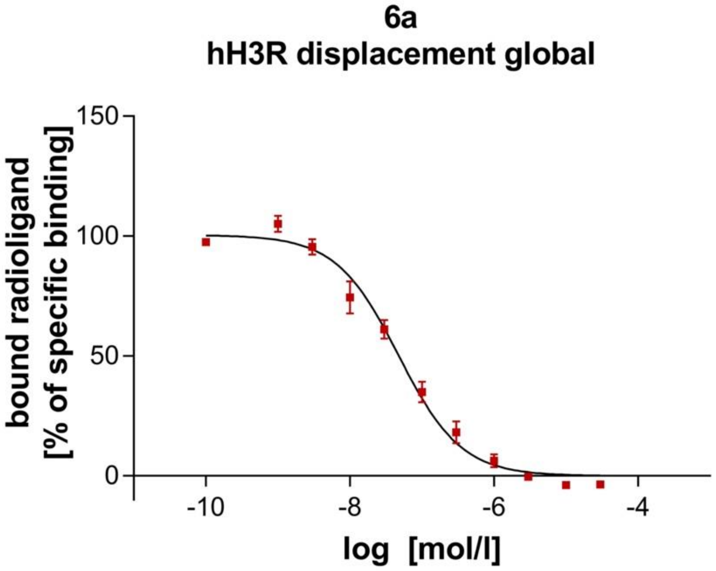

Figure S13. hH_3R competition binding curve of compounds **6a**

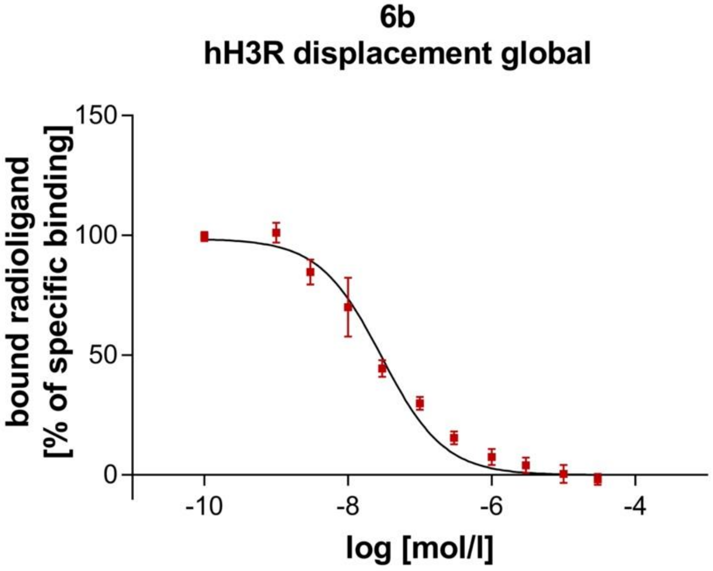

Figure S14. hH_3R competition binding curve of compounds **6b**

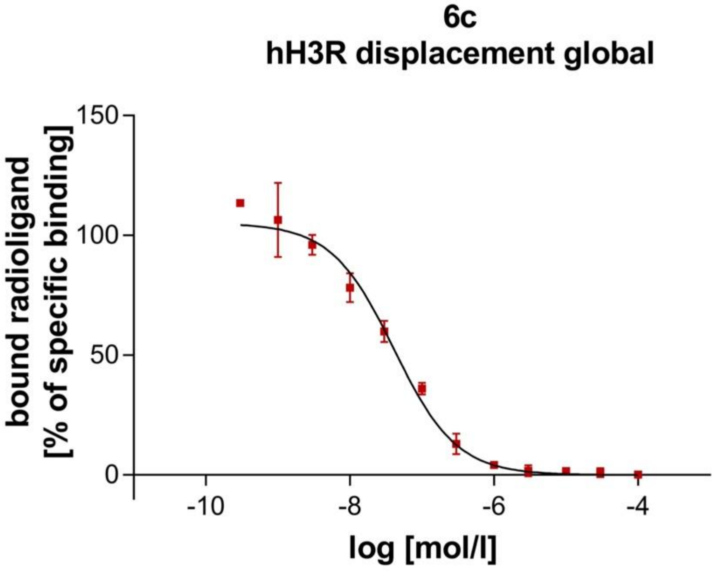

Figure S15. hH_3R competition binding curve of compounds **6c**

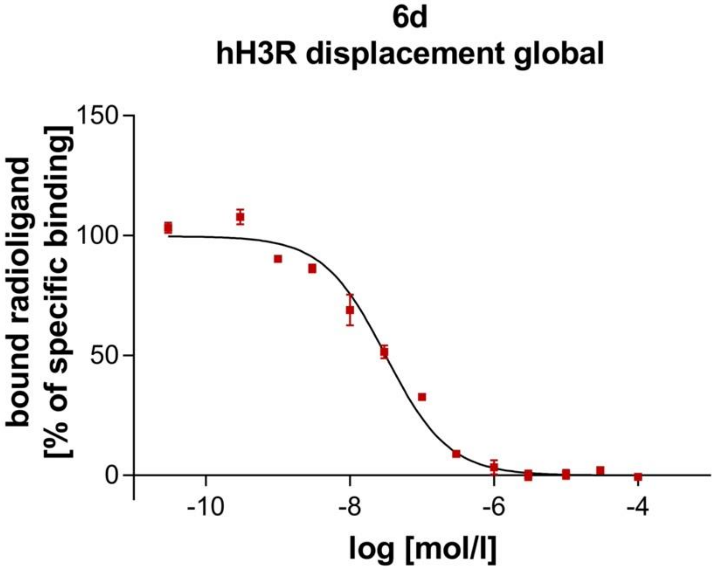

Figure S16. hH_3R competition binding curve of compounds **6d**

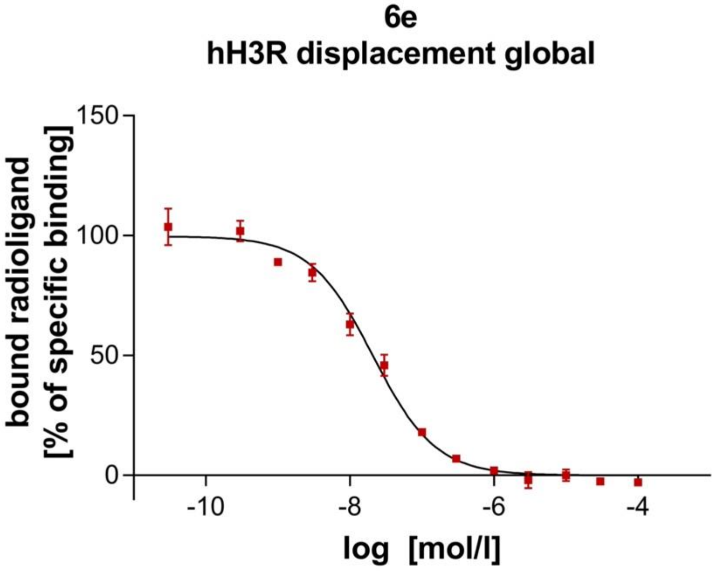

Figure S17. hH_3R competition binding curve of compounds **6e**

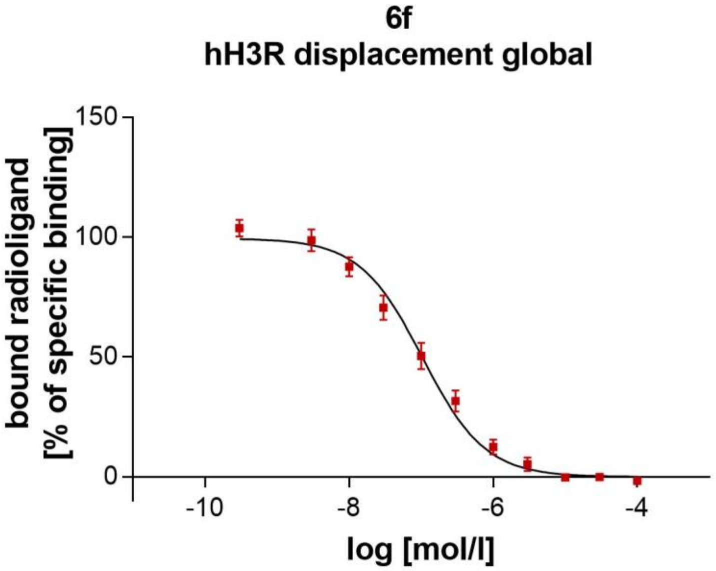

Figure S18. hH_3R competition binding curve of compounds **6f**

References

[1] K.J. Bowers, E. Chow, H. Xu, R.O. Dror, M.P. Eastwood, B.A. Gregersen, J.L. Klepeis, I. Kolossvary, M.A. Moraes, F.D. Sacerdoti, J.K. Salmon, Y. Shan, D.E. Shaw, E. Chow, H. Xu, R.O. Dror, M.P. Eastwood, B.A. Gregersen, J.L. Klepeis, I. Kolossvary, M.A. Moraes, Scalable Algorithms for Molecular Dynamics Simulations on Commodity Clusters, in: ACM/IEEE SC 2006 Conf., IEEE, New York, New York, USA, 2006: pp. 43–43. https://doi.org/10.1109/SC.2006.54.

[2] S. Genheden, U. Ryde, The MM/PBSA and MM/GBSA methods to estimate ligand-binding affinities, Expert Opin. Drug Discov. 10 (2015) 449–461. https://doi.org/10.1517/17460441.2015.1032936.

[3] P.A. Greenidge, C. Kramer, J.-C. Mozziconacci, W. Sherman, Improving Docking Results via Reranking of Ensembles of Ligand Poses in Multiple X-ray Protein Conformations with MM-GBSA, J. Chem. Inf. Model. 54 (2014) 2697–2717. https://doi.org/10.1021/ci5003735.

[4] No Title, Schrödinger Release 2021-1 Protein Prep. Wizard; Epik, Schrödinger, LLC, New York, NY, 2021; Impact, Schrödinger, LLC, New York, NY; Prime, Schrödinger, LLC, New York, NY, 2021. (n.d.).

[5] T. Kottke, K. Sander, L. Weizel, E.H. Schneider, R. Seifert, H. Stark. Receptor-specific functional efficacies of alkyl imidazoles as dual histamine H3/H4 receptor ligands. Eur J Pharmacol 2011, 654, 200-208, doi:10.1016/j.ejphar.2010.12.033.
